# Modulation of the Hypothalamic-Pituitary-Adrenal Axis by Early Life Stress Exposure

**DOI:** 10.3389/fncel.2017.00087

**Published:** 2017-04-19

**Authors:** Miranda van Bodegom, Judith R. Homberg, Marloes J. A. G. Henckens

**Affiliations:** Department of Cognitive Neuroscience, Centre for Neuroscience, Donders Institute for Brain, Cognition and BehaviourRadboudumc, Nijmegen, Netherlands

**Keywords:** HPA-axis, corticosteroids, prenatal stress, maternal separation, early social deprivation, limited nesting, match/mismatch theory, epigenetics

## Abstract

Exposure to stress during critical periods in development can have severe long-term consequences, increasing overall risk on psychopathology. One of the key stress response systems mediating these long-term effects of stress is the hypothalamic-pituitary-adrenal (HPA) axis; a cascade of central and peripheral events resulting in the release of corticosteroids from the adrenal glands. Activation of the HPA-axis affects brain functioning to ensure a proper behavioral response to the stressor, but stress-induced (mal)adaptation of the HPA-axis' functional maturation may provide a mechanistic basis for the altered stress susceptibility later in life. Development of the HPA-axis and the brain regions involved in its regulation starts prenatally and continues after birth, and is protected by several mechanisms preventing corticosteroid over-exposure to the maturing brain. Nevertheless, early life stress (ELS) exposure has been reported to have numerous consequences on HPA-axis function in adulthood, affecting both its basal and stress-induced activity. According to the match/mismatch theory, encountering ELS prepares an organism for similar (“matching”) adversities during adulthood, while a mismatching environment results in an increased susceptibility to psychopathology, indicating that ELS can exert either beneficial or disadvantageous effects depending on the environmental context. Here, we review studies investigating the mechanistic underpinnings of the ELS-induced alterations in the structural and functional development of the HPA-axis and its key external regulators (amygdala, hippocampus, and prefrontal cortex). The effects of ELS appear highly dependent on the developmental time window affected, the sex of the offspring, and the developmental stage at which effects are assessed. Albeit by distinct mechanisms, ELS induced by prenatal stressors, maternal separation, or the limited nesting model inducing fragmented maternal care, typically results in HPA-axis hyper-reactivity in adulthood, as also found in major depression. This hyper-activity is related to increased corticotrophin-releasing hormone signaling and impaired glucocorticoid receptor-mediated negative feedback. In contrast, initial evidence for HPA-axis hypo-reactivity is observed for early social deprivation, potentially reflecting the abnormal HPA-axis function as observed in post-traumatic stress disorder, and future studies should investigate its neural/neuroendocrine foundation in further detail. Interestingly, experiencing additional (chronic) stress in adulthood seems to normalize these alterations in HPA-axis function, supporting the match/mismatch theory.

## Introduction

The neuroendocrine stress response is essential for adequate responding to, coping with, and subsequent recovery from environmental threats that disrupt homeostasis (McEwen, 2007; Joëls and Baram, 2009; Sandi and Haller, 2015). Activation of the hypothalamic-pituitary-adrenal (HPA) axis provides the metabolic support for the stress response by mobilizing stored energy, suppressing the immune response, and potentiating numerous sympathetically mediated effects (de Kloet et al., 2005; Ulrich-Lai and Herman, 2009). Moreover, corticosteroids (i.e., cortisol in humans, corticosterone in rodents), the end product of the HPA-axis, easily cross the blood-brain barrier to affect brain function and thereby behavior. Although this is a highly adaptive response, aberrant corticosteroid release, e.g., as a consequence of extreme or chronic stress exposure, can be damaging to the organism and contribute to psychopathology (McKay and Cidlowski, 2003). A wealth of evidence implicates deviant HPA-axis function in stress-related mental disorders (Varghese and Brown, 2001; Faravelli et al., 2012), suggesting that proper basal and stress-induced function of the HPA-axis is of critical importance to an organism's health. Evidence for the clinical relevance of aberrant HPA-axis function has accumulated over years. Elevated basal cortisol has for example been shown predictive of the risk for depressive episodes (Goodyer et al., 2001), whereas successful antidepressant treatment is associated with the resolution of the impaired HPA-axis negative feedback (Pariante, 2006) by restoring corticosteroid receptor expression in the brain (Pariante and Lightman, 2008) that also predicts the patient's long-term clinical outcome (Pariante, 2006).

The perinatal period, characterized by elevated synaptic plasticity, reflects a critical window of brain development, during which the brain is particularly sensitive to modulating external factors such as stress (Andersen, 2003; Lupien et al., 2009). Abundant evidence suggests that stress experienced during this sensitive period can have lasting effects on an individual's ability to cope with stressful situations throughout life. Childhood adversities such as emotional, physical or sexual abuse, and neglect have been reported to result in increased arousal (Jovanovic et al., 2009), enhanced processing of negative emotional information (Pollak and Sinha, 2002; Pollak and Tolley-Schell, 2003; Pollak et al., 2009), and cognitive deficits (including impaired working memory, long-term memory, and attention; Masson et al., 2015; Geoffroy et al., 2016); all contributing to a heightened sensitivity to stress and increased risk to develop e.g., major depressive disorder (MDD), substance abuse disorders, or post-traumatic stress disorder (PTSD) (Fergusson et al., 1996; Felitti et al., 1998; Chapman et al., 2004; Faravelli et al., 2012). These observations suggest that individuals are more likely to suffer from disease as life adversity accumulates; a theory known as the cumulative stress hypothesis (McEwen, 2003). However, another prominent theory, i.e., the match/mismatch hypothesis, suggests that early life adversity may prepare an organism for exposure to similar (“matching”) adversity later in life and produce a predictive adaptive response (Gluckman et al., 2007) to optimize responses to future stressors. In line with this theory, adverse childhood events have been associated with blunted HPA-axis reactivity to acute stress experienced in adulthood (Elzinga et al., 2008). However, a mismatch between early- and later-life environments could render an organism more vulnerable to develop psychopathology (Bravo et al., 2011; Nederhof and Schmidt, 2012; Daskalakis et al., 2013; Fine et al., 2014). To understand the underlying mechanisms of vulnerability to stress-related disease and its interaction with the adult environment, it is essential to study the development of the central components of the stress system, and how this is modulated by ELS.

Here, we review existing literature describing the effects of ELS on adult HPA-axis function. As one relies on animal models to study the effects of ELS exposure prospectively, mechanistically, and in a controlled manner, this review mainly covers data from rodent studies. Like in humans, ELS in rodents has generally been shown to increase anxiety (Wigger and Neumann, 1999) and depressive symptoms (Weinstock, 2008), alter social behavior (Veenema et al., 2006; Lukas et al., 2011), impair learning and memory processes (Liu et al., 1997, 2000b), and attenuate sensorimotor gating (Ellenbroek et al., 1998; Zhang et al., 2005), seemingly in interaction with concurrent adult life stress levels (Oomen et al., 2010).

### The HPA-axis

**Figure 1:**
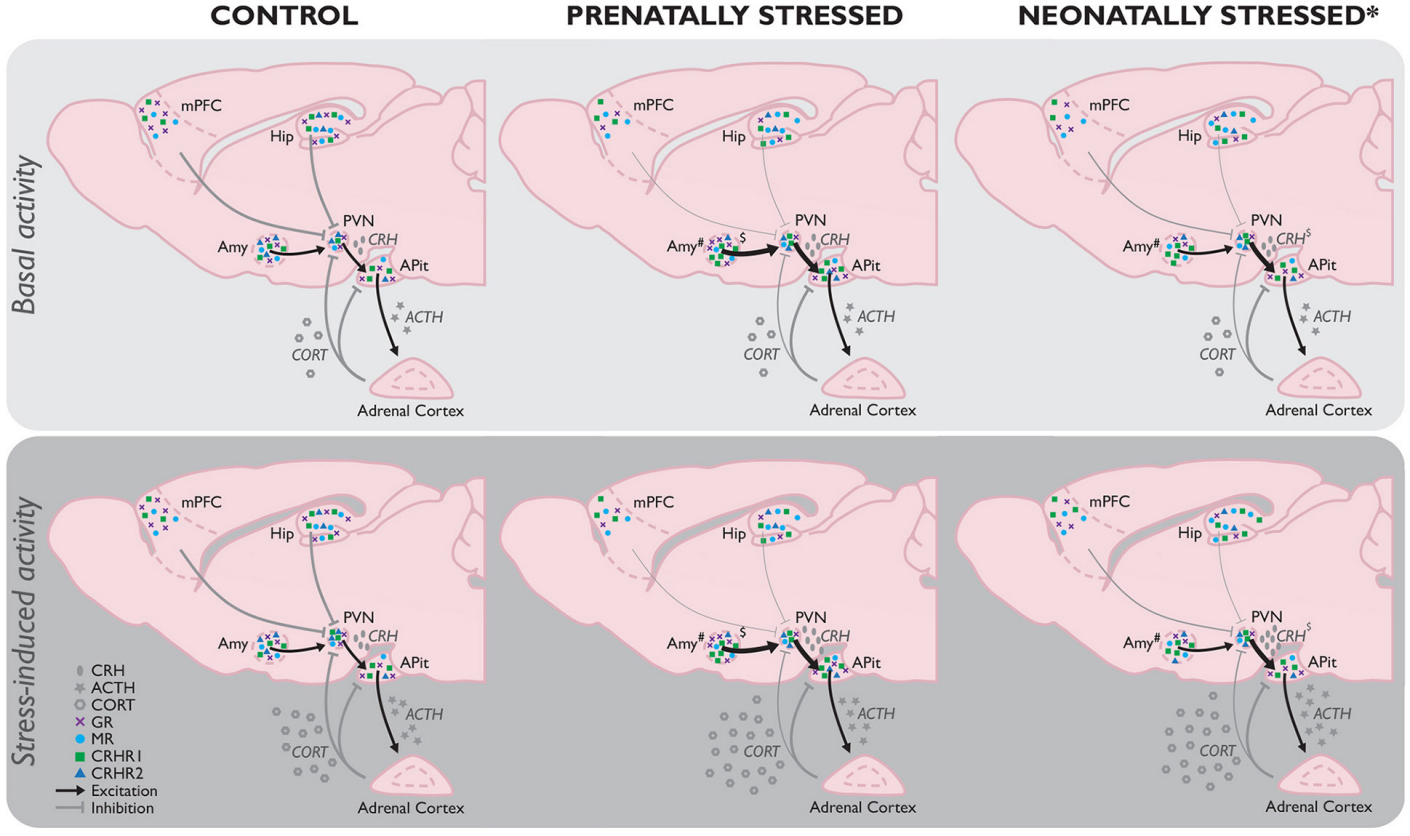
**Comparison of basal (Top)** and stress-induced **(Bottom)** HPA-axis function as a consequence of stress experienced prenatally (PS) or neonatally by maternal separation (MS; the most frequently used model for neonatal stress^*^) compared to controls without any background of early life stress. If no differences between the basal and stressed state per early life condition have been reported in literature, the absence of any difference is assumed. While the effects of MS and PS share many similarities (e.g., increased stress-induced corticosterone), there are several differences between their effects as well. Hypothalamic GR mRNA expression in the hypothalamus is unaltered in PS animals, but decreased by MS (Sutanto et al., 1996), whereas local CRHR2 mRNA expression is decreased by PS, but unaffected by MS. Amygdala GR mRNA on the other hand is increased in PS adults, and decreased in those exposed to MS. Unfortunately, because of the limited data available on the ESD model, no full picture of HPA-axis function as a consequence of this early life stressor can be constituted yet. However, initial evidence indicates ESD results in a hypo-activation of the HPA-axis, implicating a fundamentally different modulation than observed in MS and PS animals, which should be investigated in more detail in future studies. ACTH, adrenocorticotropic hormone; CORT, corticosterone; CRH, corticotrophin-releasing hormone; CRHR1, CRH receptor 1; CRHR2, CRH receptor 2; GR, glucocorticoid receptor; MR, mineralocorticoid receptor. ^*^Stress model-dependent (in LN and MS, not in ESD); ^#^subregion-specific effects; ^$^timing and stressor-dependent. For an extensive overview of findings, see Supplementary Table 1.

Upon exposure to a stressor, corticotrophin-releasing hormone (CRH) and arginine vasopressin (AVP) are secreted by the paraventricular nucleus (PVN) of the hypothalamus (Stratakis and Chrousos, 1995). CRH and AVP activate the anterior pituitary to secrete adrenocorticotropic hormone (ACTH), which in turn stimulates the adrenal cortex to produce corticosteroids, the end product of the HPA-axis (Figure 1). CRH acts primarily through CRH receptor 1 (CRHR1; Refojo and Holsboer, 2009), which is not only abundantly expressed in the anterior pituitary, but also in the prefrontal cortex, hippocampus, PVN, and basolateral amygdala (BLA); all regions involved in mediating and regulating behavioral and neuroendocrine stress responsivity. CRH also binds to a lesser extent to CRHR2, expressed predominantly in the ventromedial hypothalamus, dorsal raphe nucleus, and medial amygdala (MeA) (Steckler and Holsboer, 1999), further endorsing CRH's potency in modulating brain function. CRHR1 activation by CRH, which is not only released by the PVN, but also e.g., by CRH-expressing cells in the hippocampus and central amygdala (CeA), is generally thought mediate stress-initiation, whereas CRHR2 activation would moderate its termination, although recent work has emphasized that this dual, opposing role of CRHRs is overly simplified and highly brain-region specific (Henckens et al., 2016).

Corticosteroids easily cross the blood-brain barrier to influence brain function through the binding to two receptors: the glucocorticoid receptors (GRs) and mineralocorticoid receptors (MRs), differing both in distribution and in affinity for their ligand (Reul and de Kloet, 1985). GRs are widely expressed throughout the brain, but most abundant in the hypothalamic CRH neurons and pituitary corticotropes. MR expression is mainly restricted to the limbic areas, with highest expression levels found in the hippocampus (Sapolsky et al., 1983; Reul and de Kloet, 1985; de Kloet, 1991). The most well-known route of action of corticosteroids involves their binding to intracellularly located receptors, which upon ligand-binding translocate to the nucleus to influence gene transcription both directly through the binding of their homo/heterodimers to glucocorticoid response elements in the DNA and the recruitment of co-repressors or co-activators, or indirectly by interacting with other stress-induced transcription factors to dampen their activity (De Bosscher et al., 2003). Thereby, corticosteroid receptor binding can lead to the induction or repression of the transcription of over 200 genes that are involved in a multitude of cellular processes (Datson et al., 2001). This provides a mechanism through which corticosteroids can modulate brain maturation; initiating terminal maturation, remodeling axons and dendrites, and affecting cell survival (Meyer, 1983).

As intracellularly located MRs have a 10-fold higher affinity for corticosteroids compared to the GRs residing in the cytoplasm (Reul and de Kloet, 1985; de Kloet, 1995), they have been hypothesized to be primarily involved in the ongoing transfer of information and stability of circuits, controlling the sensitivity and the threshold of the system's response to stress. For long, the intracellular GRs were assumed to be the main players in the stress response; mediating the negative feedback control on the HPA-axis (de Kloet et al., 1993; Herman and Cullinan, 1997), normalizing neuronal activity following stress exposure, and helping the organism cope with, adapt to, and recover from stress. However, the recent discovery of MRs and GRs residing on the cell membrane, both displaying comparable affinity to the intracellular GRs (Joëls, 2008) has forced researchers to amend this rather simplistic dualistic view. Corticosteroid-binding to these membrane receptors was shown to induce rapid changes in neuronal excitability and activity through non-genomic mechanisms (Groeneweg et al., 2011) and thereby seems to contribute to an acute state of arousal and hypervigilance (de Kloet et al., 2005). This multitude of functions affected by corticosteroids suggests that alterations in corticosteroid signaling, resulting for example from early life stress (ELS), can have enormous consequences.

## HPA-axis programming by early life stress (ELS)

Because of its potent programming effects (e.g., lastingly influencing GR and MR expression levels, programming hypertension, and influencing hormone levels), excessive corticosteroid exposure to the developing brain is minimized by several mechanisms. Prenatally, fetal exposure to maternal corticosteroids is minimized by placental 11β-hydroxysteroid dehydrogenase type 2 (11β-HSD2), which throughout the entire pregnancy rapidly inactivates corticosteroids (Shams et al., 1998; Maccari et al., 2014). During late-pregnancy, the mother's HPA-axis stress response is reduced (Maccari et al., 2014), and postnatally, corticosteroid exposure to the developing brain is minimized by the stress hyporesponsive period (SHRP) (Gos et al., 2008); a period [postnatal day (PND) 1–12 in mice and PND3/4-14 in rats] characterized by both low basal ACTH and corticosteroids levels and a relative unresponsiveness to external stressors (Schmidt et al., 2003; Box 1). While not entirely identical to rodents, humans also appear to experience a period of dampened HPA-axis responses. In humans the exact duration of the SHRP is not clearly specified, but seems to occur between 6 and 12 months of age, while the human HPA-axis is still quite responsive to stressful situations up to roughly 3 months after birth (Gunnar and Donzella, 2002; Gunnar, 2003). The exact duration of this period seems to be associated with the quality of care the infant receives, with a lower quality of care resulting in premature development of corticosterone responsivity even until 15 months (Gunnar and Cheatham, 2003).

Box 1 **The developing HPA-axis.**

During pregnancy, the fetus is exposed to maternal corticosteroids which are to a great extend inactivated by placental 11β-hydroxysteroid dehydrogenase type 2 (11β-HSD2). In the third trimester (in humans, this is slightly earlier in gestation; Murphy, 1973), the fetus becomes capable of secreting corticotrophin-releasing hormone (CRH) (Fujioka et al., 1999) and adrenocorticotropic hormone (ACTH) in response to stress experienced by the mother, leading to the production of fetal corticosterone (Gunn et al., 2013). Basal corticosterone levels of the fetal rat at the final week of gestation closely resemble the basal levels found in adults (Meaney et al., 1985b; Sapolsky and Meaney, 1986; Levine, 1994). However, around birth, corticosteroid levels start to drop, resulting in low basal levels of corticosteroids and a relative unresponsiveness to external stressors early in life; the stress hyporesponsive period (SHRP) (Gos et al., 2008). During this period, pups display low basal ACTH and corticosterone concentrations and an inability to induce a ACTH/corticosterone response to stress (Schmidt et al., 2003). The pattern of CRH expression differs slightly in that the robust CRH expression decreases perinatally to ~20% of the levels observed in adulthood (Walker et al., 1986a), but increases to reach adult levels at the end of the first postnatal week (Grino et al., 1989; Baram and Lerner, 1991) and do respond to stressors during the SHRP (Dent et al., 2000a,b). The expression of arginine vasopressin (AVP) is just detectable during the third trimester, but rises in the first 4 weeks of life, reaching 70% of adult levels by postnatal day (PND) (Almazan et al., 1989). During the SHRP, glucocorticoid (GR) and mineralocorticoid receptors (MR) mRNA expression levels are slightly higher than in the prenatal brain (Yi et al., 1994), but low corticosterone levels prevent the feedback loops from functioning.

Concerning receptor expression, first CRH receptors (CRHRs) are observed in the brain from mid-gestation [~gestational day (GD)17] onwards, and reach particularly high levels early in development (stretching to >300% of adult expression levels during the first post-natal week; Insel et al., 1988; Avishai-Eliner et al., 1996). Moreover, during this stage CRHR2 is also temporarily expressed in the medial prefrontal cortex (mPFC), but has disappeared at the end of the SHRP (Eghbal-Ahmadi et al., 1998). Although, expression levels of CRHR1 in the fully developed mPFC are only moderate, and CRHR2 seems absent (Van Pett et al., 2000), initial expression levels during early development are thus much higher (Avishai-Eliner et al., 1996; Eghbal-Ahmadi et al., 1998). CRHR2 expression levels in the developing amygdala (Amy) are very subregion-dependent, as the medial and basal nuclei express this receptor by GD17, and do so fairly stable until adulthood, whereas in the cortical amygdala this receptor will not be expressed until after birth. Expression has been observed on PND3, and will increase with age (Eghbal-Ahmadi et al., 1998). In the hippocampus, CRHR1 mRNA levels increase to maximal (300–600% of adult levels) at PND6, after which levels slowly decrease (Avishai-Eliner et al., 1996). Hippocampal CRHR2 expression is observed as of PND1, and its expression remains fairly constant throughout development (Eghbal-Ahmadi et al., 1998). Prenatally, GR mRNA levels in the hippocampus, mPFC, amygdala, paraventricular nucleus (PVN), and anterior pituitary (APit) are relatively low compared to the adult situation (Bohn et al., 1994; Yi et al., 1994; Pryce, 2008) and thus, the HPA-axis has a relatively low sensitivity to negative feedback (Sapolsky et al., 1985). First GRs arise around mid-pregnancy (first in the PVN and pituitary, followed by the hippocampus) and levels rise toward the end of gestation (Matthews, 2002). Full development of GR expression only occurs after birth (when 20–50% of adult levels are observed; Sapolsky and Meaney, 1986; Levine, 1994) and continues into adulthood (Bohn et al., 1994; Pryce, 2008)^&^. MR mRNA expression also arises around mid-pregnancy (first in the pituitary, followed by the hippocampus and hypothalamus), but levels remain rather low until the last few days of gestation (Diaz et al., 1998). The concentration of MR in the hippocampus is however largely indistinguishable from adult levels by the end of the first week of life, whereas local GR levels at that time are present at only ~30% of adult levels (Meaney et al., 1985b; Sapolsky and Meaney, 1986; Sarrieau et al., 1988; Levine, 1994), making that the local ratio of MR/GR is much higher during the first weeks of life in the rodent. In the amygdala, GR and MR expression increases gradually over development in a region-specific manner (Yi et al., 1994; Diaz et al., 1998).

Upon adulthood, corticosteroid levels have increased significantly, as have GR and MR mRNA expression in the hippocampus, mPFC, amygdala, and PVN (Bohn et al., 1994; Yi et al., 1994; Pryce, 2008). CRH secreted by the pituitary stimulates the secretion of ACTH, which in turn stimulates the production and secretion of corticosterone by the adrenal glands, which is now capable of taking part in the fully functional feedback loops of the HPA-axis. A critical period in HPA-axis development not covered by this review is adolescence (extensively reviewed elsewhere e.g., McCormick et al., 2010; Eiland and Romeo, 2013). Interestingly, recent investigations have indicated that (pharmacological) interventions targeting the GR during this period are able to revert the effects of ELS on the brain (Arp et al., 2016; Loi et al., 2017), making that this period—and its exact HPA-axis characteristics—deserves further study.

^&^Development in the mouse brain differs slightly from that in the rat, with e.g., GR expression in the hippocampus not being observable until after birth (Noorlander et al., 2006), suggesting species-specific maturation of the HPA-axis.

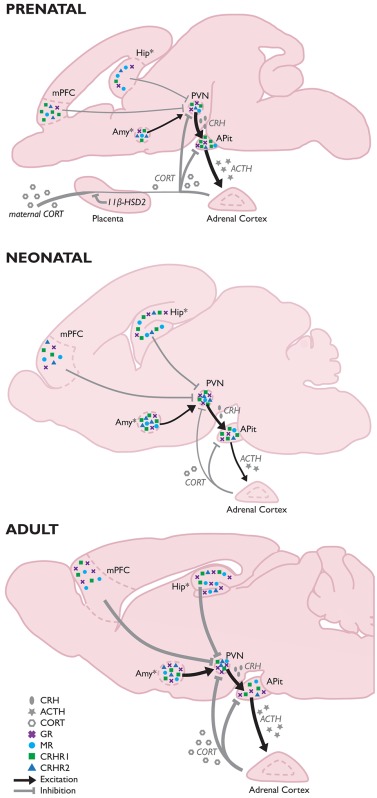

However, these mechanisms cannot prevent corticosteroid exposure entirely, allowing ELS to affect brain and HPA-axis development. While 11β-HSD2 buffers corticosterone exposure to the fetus, a portion of it does pass the placenta, where it not only increases fetal exposure directly but also indirectly by inducing fetal HPA-axis activation (provided the fetal HPA-axis is functional yet; Ohkawa et al., 1991; Fujioka et al., 1999; Seckl, 2008). Moreover, repeated exposure of the mother to stress reduces 11β-HSD2 activity (Mairesse et al., 2007), further contributing to increased fetal corticosteroid exposure, reaching levels high enough to cause alterations in fetal programming (e.g., by influencing GR and MR expression levels and inducing increased HPA-axis activity; Levitt et al., 1996). Increased corticosteroid exposure seems to critically mediate these ELS effects, as they are prevented by adrenalectomy and hormone replacement in the dams (Barbazanges et al., 1996). Shortly after birth, prolonged stress exposure, e.g., by long separation periods (3 h or more each day) from the mother, can cause the neonate to emerge from the SHRP; increasing activity of the PVN (Smith et al., 1997) and elevating levels of basal and stress-induced corticosterone (Stanton et al., 1988). Short separation periods (3 min–3 h) seem to be insufficient to do so, but when repeated daily, also induce sensitization of the neonate's corticosterone stress response and adrenal growth (Levine et al., 1991; D'Amato et al., 1992; McCormick et al., 1998; Schmidt et al., 2004).

Thereby, ELS is able to “imprint” or “program” an organism's neuroendocrine, neural and behavioral responses to stress. Although the exact underlying mechanisms by which ELS establishes these life-long effects still need to be resolved, research focuses along two complementary lines. Firstly, ELS during critical stages in brain maturation may disrupt specific developmental processes (by altered neurotransmitter exposure, gene transcription, or neuronal differentiation), leading to aberrant neural circuit function throughout life (Chen and Baram, 2016). Evidence for these mechanisms is derived from observations that corticosteroids *in vitro* decrease the rate of cell proliferation by preventing progression through the cell cycle (Fanger et al., 1987; Vintermyr et al., 1989; Hatakeyama et al., 1991; Sánchez et al., 1993), suggesting that endogenous corticosteroids play a role in differentiation and maturational events during late fetal brain development, promoting the transition between a proliferative and a differentiating stage by directly inhibiting cell division as well as activating the expression of specific genes characteristic of the differentiated mature phenotype. Secondly, ELS may induce modifications of the epigenome which lastingly affect brain function (Babenko et al., 2015). Briefly, epigenetics refers to mechanisms by which the environment interacts with the genome by the modification of chromatin structure or control of mRNA translation (Silberman et al., 2016). DNA methylation, post-translational histone modifications (methylation, phosphorylation, acetylation) and noncoding RNA activity are among the most studied epigenetic mechanisms that regulate gene expression. These epigenetic modifications are inducible, stable, and yet reversible, constituting an important emerging mechanism by which transient environmental stimuli can induce persistent changes in gene expression and ultimately behavior (Zovkic et al., 2013). Prenatal stress was for example shown to alter characteristic brain miRNA profiles and affect transcriptomic brain profiles in the offspring, including genes related to development, axonal guidance and neuropathology (Zucchi et al., 2013). Furthermore, increased DNA methylation of the *Hsd11b2* gene promoter in the placenta was found (together with an increase in DNA methyltransferase DNMT3a mRNA levels) as a consequence of repeated stress exposure of the mother, which is held responsible for the reduction of *Hsd11b2* mRNA expression and activity observed following repeated stress (Jensen Peña et al., 2012). Moreover, in the fetal hypothalamus, prenatal stress was found to decrease methylation within the *Hsd11b2* promoter and increase methylation at sites within exon 1 of the gene, but these differences did not translate into differential mRNA expression levels of the gene.

To improve understanding of the life-time consequences of these ELS-induced mechanisms and their potential contribution to psychopathology, we here review the effects of ELS on the functional and structural integrity of the HPA-axis' endocrine glands, expression levels of neuroendocrine and growth hormones and neurotransmitters, as well as their receptors in several of the key brain regions regulating HPA-axis activity (i.e., the amygdala, hippocampus, and prefrontal cortex), and interpret their (mal)adaptive nature under either matching or non-matching adult life circumstances. Unless specified otherwise, the discussed data apply to animals in adulthood.

### ELS induction

To study the effects of *prenatal stress* (PS) exposure on the offspring, dams are most frequently stressed by physical restraint (Lemaire et al., 2000; Mandyam et al., 2008; Belnoue et al., 2013; de Souza et al., 2013; Madhyastha et al., 2013; Xu et al., 2014) or immobilization of the limbs (Liaudat et al., 2015), often for multiple times a day. Alternatively, exposure to foot shocks (Estanislau and Morato, 2005, 2006), hypoxia (Fan et al., 2009; Wang X. et al., 2013), or multiple variable stressors over the course of multiple days (Lee et al., 2007; Fan et al., 2009; Zohar and Weinstock, 2011) are applied. These stressors are most commonly applied during the second half of pregnancy, a critical moment in fetal brain development when the differentiation of several key regions in the regulation of the stress response initiates (i.e., hypothalamus, amygdala, and hippocampus; see Bayer et al., 1993 for a review concerning the human compared to the rat brain development), and ranges between a single stressor to repeated stress exposure until birth (Welberg et al., 2001; Fan et al., 2009).

To induce neonatal stress, *maternal separation* (MS) is often used, i.e., the temporary separation of the dam from her pups, which models maternal neglect. In the variant of *early social deprivation* (ESD), the pups are isolated from both their mother and littermates and temporarily housed in a novel environment (Sandi and Haller, 2015). The separation duration, frequency and its timing, vary amongst studies, influencing their ultimate effect on HPA-axis function and brain development in the neonate. A third, relatively recent, neonatal stress model is the *limited nesting model* (LN; Rice et al., 2008), in which dams are housed in a cage with only limited nesting (or bedding) material available. The absence of sufficient material to build a proper nest induces chronic stress in the dam (Ivy et al., 2008), disrupts maternal behavior and fragments the dam's care for her pups (Rice et al., 2008), as opposed to the separation models, which typically cause a temporary increase in the dam's care upon their reunion (e.g., Pryce et al., 2001, see Box 2). As maternal care (mostly through feeding and tactile stimulation) suppresses pups' HPA-axis activity (e.g., Macrì et al., 2008), the LN model raises neonatal corticosteroid levels as well (Gilles et al., 1996; Avishai-Eliner et al., 2001) by being a chronic stressor [whereas MS and ESD are more acute (recurrent) neonatal stressors]. Due to its resemblance of impoverished maternal care in human situations, the LN paradigm is a valuable addition to the earlier models of neonatal stress.

Box 2 **Stress-induced variations in maternal care and their impact.**

Maternal care has been shown to be a critical modulator in the effects of early life stress (ELS) on the developing offspring. Being either essential to the manipulation [e.g., in limited nesting (LN)] or an “unwanted side effect” [e.g., in the case of prenatal stress (PS)], the effect of the stressor on maternal behavior is a crucial denominator of its eventual effects on the offspring. Enhanced maternal care (arch back nursing and licking and grooming behavior) induced by neonatal handling of pups induces physiological responses related to reduced fearfulness, and improved emotional, behavioral, and neuroendocrine stress responses (see Francis et al., 1999 for a review of this work). Handled animals show reduced basal corticotrophin-releasing hormone (CRH) expression (Plotsky and Meaney, 1993; Plotsky et al., 2005), and reduced CRH receptor 1 expression in the paraventricular nucleus and locus coeruleus (contributing to the noradrenergic drive induced by stress; Plotsky et al., 2005), which is joined by increased negative feedback sensitivity to corticosteroids, related to increased hippocampal and frontal cortex glucocorticoid receptor (GR) expression (Meaney et al., 1985a; Sarrieau et al., 1988). Studies investigating the natural variation in nursing behavior observed similar changes in the offspring of high licking and grooming mothers (Liu et al., 1997), and showed that corticosterone responses to acute stress as well as hippocampal GR mRNA and hypothalamic CRH mRNA expression correlated with the nursing behavior of the dam. Moreover, maternal care was recently shown to exert major influence on the DNA methylation, histon acetylation and gene expression across large genomic regions (covering the *NR3C1*) in the hippocampi of adult animals (McGowan et al., 2011). Differences in transcription occurred in the context of hyperacetylation and hypomethylation of promoters and hypermethylation of exons. These studies suggest that the behavior of the mother toward her pups can program neuroendocrine responses to stress in adulthood. In the ELS paradigms altered care is crucial for many of the observed stress effects, as demonstrated by the use of foster-studies (Maccari et al., 1995; Huot et al., 2004; de Souza et al., 2013). As PS can also induce alterations in maternal behavior (de Souza et al., 2012; St-Cyr and McGowan, 2015), these paradigms may in fact model the effects of a both prenatal and neonatal stressful environment instead of looking at PS in isolation. Although, altered maternal care as a consequence of stress during pregnancy is not always observed (e.g., in Lee et al., 2007), cross-fostering studies emphasize the impact on the postnatal environment (i.e., maternal care). The effects of PS in terms of the stress-induced corticosterone response, anxiety, aggression, and social memory differed significantly in pups raised by either control foster mother, non-related stressed mothers, or their biological stressed mother (Maccari et al., 1995; de Souza et al., 2013).

In case of the LN model alterations in maternal behavior (Gilles et al., 1996; in terms of erratic and fragmented nurturing behavior) are in fact sufficient for long-term stress effects in the offspring (Brunson et al., 2005; Rice et al., 2008; Dalle Molle et al., 2012; Gunn et al., 2013). Similarly, maternal care seems to critically mediate the effects of maternal separation (MS) and early social deprivation (ESD), as in these paradigms not only the pups are stressed by the absence of their mother, but the mothers as well. The amount of stress experienced by the dam (and the compensatory care she can perform) however, greatly varies amongst the separation procedures implemented, influencing her behavior toward the litter. Besides the obvious differences in separation frequency and duration, some MS studies leave the litter in the home cage (Sutanto et al., 1996; Vázquez et al., 2003), removing the mum, whilst others place the litter in a new, clean cage (Aisa et al., 2008). Understandably, the exposure to a novel environment might cause additional stress in either the dam or pups, causing variable stress levels between studies. Amongst ESD studies similar variations arise, as they either allow the mother to keep part of her litter by her side (Barna et al., 2003) or separate her from all her pups (Irles et al., 2014), which is evidently more stressful. Potentially depending on the amount of stress experienced by the dam, temporary compensatory increases in nurturing behavior following the reunion with her pups are also observed (Macrì et al., 2008), which might modulate the impact of the stressful separation on the offspring.

### Effects on the endocrine glands and their output

#### Hypothalamus

The hypothalamic PVN develops to a great extent prenatally, and seems to be part of a functioning HPA-axis from the third trimester onwards [gestational day (GD) 17 in rats], when regional CRH mRNA responses are observed to maternal stress (Fujioka et al., 1999). The observation that CRH synthesis and mRNA expression in the fetal hypothalamus are not yet regulated by corticosteroids until the end of the first postnatal week (Grino et al., 1989; Baram and Schultz, 1992; Yi and Baram, 1993), and local CRHR expression levels are particularly high early in development (Insel et al., 1988), implicates an important role for the stress-induced elevations in CRH signaling mediating the effects of ELS on PVN function.

On the structural level, previous work has indicated that PS increases apoptosis in the fetal hypothalamus (Fujioka et al., 1999; Tobe et al., 2005), but decreases apoptosis in adulthood (Baquedano et al., 2011). Although MS was not found to affect local neuronal density during the SHRP, it increased neuronal density afterwards, which was joined by decreased levels of apoptosis-stimulating proteins and enzymes, whilst cell survival-stimulating protein levels were increased (Irles et al., 2014). These data indicate that ELS influences the structural reorganization of the PVN throughout development, and thereby likely alters its role in HPA-axis regulation.

On the functional level, the effects of ELS on both basal and stress-induced CRH release by the PVN seem to heavily depend on the precise developmental period affected by ELS, the stressor applied, and the age at which the effects are assessed (see Table 1 for an overview of findings). Moreover, the effect of ELS on local CRH signaling might be sex-specific, as PVN CRHR1 mRNA and protein levels were reported to be increased as a consequence of PS in males, but decreased in females (Fan et al., 2009; Wang X. et al., 2013; see Box 3 for an overview of sex-specific modulatory effects of ELS). However, these findings are in contrast with another study reporting no ELS-effects on PVN CRHR1 expression in either sex (Zohar and Weinstock, 2011). As CRHR1-activation in the PVN has been associated with anxiogenic effects (Fan et al., 2013), elevated CRHR1 levels in PS males could underlie the anxious behavioral profile resulting from ELS (Huot et al., 2002; Kalinichev et al., 2002; Daniels et al., 2004; Rees et al., 2006; Aisa et al., 2007; Trujillo et al., 2016). In contrast to potentially increased PVN CRHR1 levels, local CRHR2 expression is unchanged or reduced in both sexes as a consequence of PS (Fan et al., 2009; Zohar and Weinstock, 2011; Wang X. et al., 2013) or MS (Bravo et al., 2011; O'Malley et al., 2011).

**Table 1:** **CRH mRNA expression in the PVN both under basal conditions and in response to stress in ELS animals compared to non-stressed controls**.

**Period**	**Stress paradigm**	**Duration**	**Age at testing**	**Basal CRH**	**Stress-induced CRH**	**Species (strain)**	**References**
GD1–21	CBX	daily	Adult	↑^♂^		Rats (Wistar)	Welberg et al., 2000
GD1–21	Hypoxia and/or restraint	4 h	Adult	↑^♂^^*^		Rats (SD)	Fan et al., 2009
GD1–21	Hypoxia	4 h	Adult	↑^♂^/–^♀^		Rats (SD)	Wang X. et al., 2013
GD4–10	Defeat or restraint^#^	45 or 60 min	Adult	↑^♀^		Rats (Wistar)	Bosch et al., 2007
GD11–18	Defeat	1 h					
GD9–20	Restraint	6 h	Adult	–^♂^^*^		Mice (ICR)	Chung et al., 2005
GD13–21	Variable	daily	Adult	–^♂^/↑^♀^	–^♂^/↑^♀^	Rats (Wistar)	Zohar and Weinstock, 2011
GD16–20	Defeat	10 min	Adult	–	↑	Rats (SD)	Brunton and Russell, 2010
PND2–9	LN		PND9	↓		Rats (SD)	Avishai-Eliner et al., 2001
PND2–9	LN		PND9	↓		Mice (C57BL/6J)	Rice et al., 2008
PND3	MS	24 h	PND20	–		Rats (SD-LE hybrids)	van Oers et al., 1997
PND3	MS	24 h	PND20	–	↑	Rats (SD-LE hybrids)	van Oers et al., 1998b
PND5	MS	24 h	PND 6	↓	↓	Rats (SD-LE hybrids)	Dent et al., 2000a
PND7	MS	24 h	PND20	–		Rats (SD-LE hybrids)	van Oers et al., 1997
PND8	MS	24 h	PND9	–	–	Rats (SD)	Avishai-Eliner et al., 1995
PND8	ESD	24 h	PND9	–	–	Rats (SD)	Avishai-Eliner et al., 1995
PND11	MS	24 h	PND12	↓	↓	Rats (SD-LE hybrids)	Smith et al., 1997; van Oers et al., 1998a
PND11	MS	24 h	PND12	–	↓^$^	Rats (SD-LE hybrids)	Dent et al., 2000a
PND11	MS	24 h	PND20	–		Rats (SD-LE hybrids)	van Oers et al., 1997
PND11	MS	24 h	PND20	–	↓	Rats (SD-LE hybrids)	van Oers et al., 1998b
PND17	MS	24 h	PND18	–	↑	Rats (SD-LE hybrids)	Dent et al., 2000a
PND19	MS	24 h	PND20	↓	↓	Rats (SD-LE hybrids)	Smith et al., 1997
PND1–14	ESD	4 h	Adult	–		Rats (Ficher)	Rüedi-Bettschen et al., 2006
PND2–8	MS	15 min	Adult	↓		Rats (SD)	Korosi et al., 2010
PND2–9	LN		Adult	↓^♂^		Mice (C57BL/6J	Rice et al., 2008
PND2–9	LN		Adult	–^♂^		Mice (129S2/Sv × C57BL/6J)	Wang et al., 2012
PND2–13	MS	4 h	Adult	–	–	Rats (SD)	Chen et al., 2012
PND2–14	MS	3 h	Adult	↑^♂^	↑^♂^	Rats (LE)	Plotsky and Meaney, 1993
PND2–14	MS	3 h	Adult	↑^♂^		Rats (LE)	Plotsky et al., 2005
PND2–21	MS	3 h	Adult	↑^♀^		Rats (Wistar)	Aisa et al., 2008
PND3	MS	24 h	Adult	↓^♂^		Rats (Brown Norway)	Workel et al., 2001
PND3–15	MS	3 h	Adult	–^♂^		Rats (LE)	Slotten et al., 2006
PND9	ESD	24 h	Adult		–	Rats (n.s.)	Barna et al., 2003

*If no sex is specified, results apply to both males and females. ↓ Indicates a significant decrease, ↑ a significant increase, and – no significant difference in corticotrophin-releasing hormone mRNA expression. Stressors are applied daily for the indicated period*.

♂ Results apply to males;

♀ results apply to females;

# applied on alternating days;

* CRH protein expression;

$ *response levels are unaffected but the stress response is shorter-lasting; CRH, corticotrophin-releasing hormone; ESD, early social deprivation; GD, gestational day; LE, Long Evans rats; LN, limited nesting; MS, maternal separation; n.s., not specified; PND, postnatal day; SD, Sprague Dawley rats*.

Box 3 **Sex-differences in ELS effects.**

There is a considerable sex-bias in the prevalence of stress-related mental disorders linked to early life adversity (Tolin and Foa, 2006; American Psychiatric Association, 2013). The increased susceptibility of women to stress-related psychopathology may be may be (partially) mediated by sex-specific (vulnerable) responses to early life stress (ELS). Females experiencing trauma, physical abuse, or maternal distress during infancy show higher rates of depression, anxiety, and post-traumatic stress disorder than males (Baker and Shalhoub-Kevorkian, 1999; MacMillan et al., 2001; Pitzer et al., 2011). Sex also seems to be a significant modulator of the relationship between childhood adversity and HPA-axis activity later in life. Exposure to early trauma is associated with higher basal corticotrophin-releasing hormone (CRH) levels in women, but lower levels in men, whereas severe trauma is linked to an increased response to a CRH challenge in men only (DeSantis et al., 2011). There is also evidence from rodent studies indicating sex-specific effects of ELS on neuroendocrine function. Prenatally stressed (PS) females were shown to display a higher peak corticosterone plasma levels to stress in adulthood compared to males (Brunton and Russell, 2010). They display increased fetal paraventricular nucleus (PVN) apoptosis in response to acute immobilization (Tobe et al., 2005), as well as higher basal PVN CRH (though inconsistently) and arginine vasopressin mRNA expression levels (Brunton and Russell, 2010; Zohar and Weinstock, 2011), and higher basal adrenocorticotropic hormone plasma levels as a result of PS compared to males. However, females do not display a significantly higher acute stress-induced increase in POMC mRNA expression in the anterior pituitary compared to non-stressed controls, while males do (Brunton and Russell, 2010). CRH receptor 2 mRNA expression in the basomedial amygdala is increased in PS females, whereas it is decreased in PS males. Meanwhile, effects of PS on CRH receptor 1 mRNA expression in the amygdala appear to be both sex- and subregion-specific; mRNA expression was found to be significantly elevated in the central amygdala and basolateral amygdala of males as a consequence of PS, but in the medial amygdala of females (Brunton et al., 2011). PS induces reductions in adult neurogenesis in males (Lemaire et al., 2000; Mandyam et al., 2008; Morley-Fletcher et al., 2011; Belnoue et al., 2013; Madhyastha et al., 2013), but does not seem to affect neurogenesis in females (Mandyam et al., 2008; Zuena et al., 2008), which might be related to overall lower basal levels of neurogenesis in adult females (Mandyam et al., 2008; Oomen et al., 2009). Some of these effects might be related to sex-specific epigenetic regulation of gene expression during development. PS was shown to cause significant elevations in DNA methyltransferase 1 expression in the placenta of females, but not in males, but only male brains displayed reduced hippocampal glucocorticoid receptor and increased amygdalar CRH expression, which was related to changes in *Crh* and *NR3C1* methylation (Mueller and Bale, 2008).

Neonatal stress studies have indicated similar sex-differences. Female rats were found to overall display higher basal plasma corticosterone levels than males, but these were reduced by maternal separation (MS) (Slotten et al., 2006). Similarly, basal corticosterone levels of female mice have been found to be reduced as a consequence of limited nesting (LN), in contrast to increased basal level in males (Arp et al., 2016). However, such sex differences are not consistently found (**Table 3**). MS also affects adult neurogenesis differently in males and females, depending on the age of assessment. At the age of weaning, male rats were found to display increased neurogenesis, whereas MS female rats displayed decreased levels (Oomen et al., 2009), but these effects changed at adulthood, when neurogenesis was found to be reduced in MS and LN males (Oomen et al., 2010; Lajud et al., 2012; Naninck et al., 2015), but unaffected in females (Oomen et al., 2011; Naninck et al., 2015).

Unfortunately, most of what is known about the effects of ELS on brain maturation is derived from studies using male individuals, particularly in rodent research, illustrating the necessity of the more thorough investigation of sex differences in neuroscience research (Beery and Zucker, 2011). Moreover, one should take the oestrous cycle phase at the moment of testing of females into account, as it seems to be an important modulating factor when assessing the effects of ELS (Romeo et al., 2003), but is often ignored.

In addition to CRH, hypothalamic AVP has subtle stimulating effects on ACTH secretion as well (Gillies et al., 1982) and potentiates the effects of CRH (Giguere and Labrie, 1982; Gillies et al., 1982; Lolait et al., 2007). MS has been found to increase local basal AVP mRNA expression at PND14 (Vázquez et al., 2003), PND21 (Zhang et al., 2012), and PND35 (Veenema and Neumann, 2009), and to elevate local stress-induced AVP mRNA levels at PND6 and PND12 in rats (Dent et al., 2000a), whereas it increases stress-induced *fos* expression in AVP-positive PVN cells (Zhang et al., 2012). In adults, local stress-induced AVP mRNA and protein levels are higher in both PS (Brunton and Russell, 2010) and MS (Veenema et al., 2006, 2007) offspring compared to controls, although effects might be sex- and stressor-specific (Desbonnet et al., 2008; Brunton and Russell, 2010). Like for CRH, effects of ELS on basal PVN AVP expression are rather heterogeneous. While PS exposure does not affect basal AVP mRNA expression in the male PVN (Lee et al., 2007; Brunton and Russell, 2010), it increases local levels in the females (Bosch et al., 2007; Brunton and Russell, 2010). The number of local AVP-expressing cells has however been found to be decreased due to PS (de Souza et al., 2013) in both sexes. Moreover, effects of PS on AVP expression might be depending on genetic background. Basal PVN AVP mRNA expression in rats bred for low levels of anxiety-related behavior (LAB) were found to be lower compared to rats bred for high levels of anxiety-related behavior (HAB), but PS increased AVP mRNA expression in the LAB rats to levels observed in HAB rats, the latter being not affected by PS (Bosch et al., 2006). Neonatal stress was found to either not affect (Veenema et al., 2006), increase (Veenema et al., 2007; Desbonnet et al., 2008; Murgatroyd et al., 2009; Zhang et al., 2012), or decrease (in females) (Desbonnet et al., 2008) basal expression levels compared to unstressed controls. In one of the studies, increased AVP signaling induced by neonatal stress exposure was associated with a sustained DNA hypomethylation of the *Avp* gene in the PVN, and turned out to critically mediate the observed hypersecretion of corticosterone and alterations in passive stress coping and memory observed in the offspring (Murgatroyd et al., 2009). However, further research seems necessary to elucidate the exact effects of ELS on AVP signaling.

In the adult PVN, GR mRNA has been localized to cells expressing CRH (Swanson and Simmons, 1989), where GR moderates the glucocorticoid-mediated negative feedback on the HPA-axis by regulating CRH gene expression (Majzoub et al., 1993). Both prenatal exposure to exogenous corticosterone and PS have been shown to decrease local GR expression (Bingham et al., 2013). Inhibition of 11β-HSD2 during pregnancy, raising prenatal corticosterone exposure, also induced reduced GR mRNA expression in the PVN (while it locally increased CRH mRNA levels; Welberg et al., 2000), whereas MS attenuated GR-binding in young rats and decreased GR mRNA levels in adult (Arnett et al., 2015) and senescent animals (Workel et al., 2001). These findings suggest that ELS attenuates HPA-axis regulation at the level of the PVN by reducing GR-mediated negative feedback.

#### Anterior pituitary

Both corticosteroid and CRH receptors are present in the pituitary from the third trimester onwards (Insel et al., 1988). In contrast to the PVN, pro-opiomelanocortin (POMC; the precursor for ACTH) transcription is already stimulated by CRH and inhibited by corticosteroid administration at this age, implicating functional receptors and local negative feedback regulation well before birth (Scott and Pintar, 1993). However, soon after birth, the pituitary shows a time-limited, reduced response to CRH, which could either be the result of a reduced sensitivity to CRH (Dent et al., 2000b) (although CRHR expression is high at that time) or a reduction in the size and number of ACTH-secreting cells in the pituitary (Sapolsky and Meaney, 1986). Exaggerated negative feedback-sensitivity to corticosteroids (Walker et al., 1986b) might further contribute to this non-responsiveness, but this cannot be readily explained by altered corticosteroid receptor expression levels (which are relatively stable prenatally, and only slowly increase after birth to reach adult levels; Keller-Wood et al., 2006).

Similar to the PVN, the pituitary of PS animals is characterized by decreased cell proliferation and cell death in adulthood (Baquedano et al., 2011). Basal ACTH and POMC expression levels seem to be rather unaffected by ELS in the adult offspring (see Table 2 for an overview of findings). While increased basal ACTH levels have been reported for PS females, ACTH plasma levels seem to be unaffected by neonatal stress. Interestingly, though basal POMC mRNA levels are not influenced by PS (Brunton and Russell, 2010), they are elevated by MS (Murgatroyd et al., 2009), associated with an enduring hypomethylation of the POMC gene (Wu et al., 2014), indicating alterations in ACTH turnover. Concerning stress-induced responses, PS seems to increase the POMC mRNA (in males) and ACTH response to stress (Fan et al., 2009; Brunton and Russell, 2010), at least partially by increasing CRHR expression in the anterior pituitary (Fan et al., 2009). The effects of neonatal stressors on stress-induced ACTH release however seem to again greatly depend on the type of stressor, its timing and duration, and the age of the animal at which the effects are assessed (Table 2). Generally, MS for 24 h both before the onset of and early with in the SHRP is found to increase offspring's ACTH plasma levels in response to stress. However, MS during the second half of the SHRP increases ACTH response to stress only if tested *during* the SHRP (Smith et al., 1997; van Oers et al., 1998b), whereas it reduces ACTH stress responses measured at an older age (Smith et al., 1997; van Oers et al., 1997, 1998b). AVP seems to play an important role in mediating these effects, as the increase in ACTH levels as a consequence of 24 h MS on PND9 was not observed in AVP deficient animals (Zelena et al., 2015). Since AVP deficiency or AVPR1b antagonist pretreatment diminished ACTH responses to stress only in pups but no longer in adults (Zelena et al., 2011), AVP seems to be particularly important in regulating ACTH-secretion in the neonate. LN seems to reduce ACTH stress-induced responses during the SHRP, though data is limited. Both multiple-day MS and 24 h MS during the SHRP seem to increase ACTH stress responses in adulthood, but not consistently. ESD does not exert any obvious effect (Table 2). Stressor- and age-dependent alterations in local CRHR binding capacity may contribute to the diversity of these effects. ESD and LN are for example found to reduce CRHR binding capacity (Ladd et al., 1996; Avishai-Eliner et al., 2001), and thereby limit the ACTH-releasing potential of CRH.

**Table 2:** **ACTH plasma levels in ELS animals as compared to non-stressed controls**.

**Period**	**Stress paradigm**	**Duration**	**Age at testing**	**Basal ACTH**	**Stress-induced ACTH**	**Species (strain)**	**References**
GD1–21	Hypoxia and/or restraint	4 h	Adult	↑^♂^		Rats (SD)	Fan et al., 2009
GD4–10	Defeat or restraint^#^	45 or 60 min	Adult	–^♀^	–/↑^♀^^@^	Rats (Wistar)	Bosch et al., 2007
GD11–18	Defeat	1 h					
GD9–20	Restraint	6 h	Adult	–^♂^	–^♂^	Mice (ICR)	Chung et al., 2005
GD15–19	Restraint	3 × 45 min	Adult	–^♂^/↑^♀^	↓^♂^^$^/↑^♀^	Rats (LE)	McCormick et al., 1995
GD15–21	Restraint	20 min	Adult	↑^♀^		Rats (Wistar)	Pérez-Laso et al., 2008
GD16–20	Defeat	10 min	Adult	–^♂^/ –/↑^♀^^∧^	↑	Rats (SD)	Brunton and Russell, 2010
PND1–10	MS	3 h	PND42	↑^♂^		Mice (C57Bl/6N)	Wu et al., 2014
PND2–9	LN		PND10	–	↓	Rats (SD)	McLaughlin et al., 2016
PND2–14	MS	5 h	PND30	–^♀^	–^♀^	Rats (SD)	Rees et al., 2006
PND2–14	ESD	5 h	PND30	–^♀^	–^♀^	Rats (SD)	Rees et al., 2006
PND3	MS	24 h	PND4	–	↑	Rats (SD-LE hybrids)	van Oers et al., 1998b
PND3	MS	24 h	PND20	–	↑	Rats (SD-LE hybrids)	van Oers et al., 1997, 1998b
PND4	MS	24 h	PND5	↑	↑/–^&^	Mice (CD1 + C57Bl/6J)	Daskalakis et al., 2014
PND5	MS	24 h	PND6	–	↑	Rats (SD-LE hybrids)	Dent et al., 2000a
PND6	MS	24 h	PND7	–	↑	Rats (Wistar)	Vázquez et al., 1996
PND7	MS	24 h	PND20	–	↓	Rats (SD-LE hybrids)	van Oers et al., 1997
PND9	MS	24 h	PND10	–	↑	Rats (Wistar)	Vázquez et al., 1996
PND11	MS	24 h	PND12	–	↑	Rats (SD-LE hybrids)	Smith et al., 1997; van Oers et al., 1998a,b; Dent et al., 2000a
PND11	MS	24 h	PND16	–	↓	Rats (SD-LE hybrids)	van Oers et al., 1997
PND11	MS	24 h	PND20	–	↓	Rats (SD-LE hybrids)	van Oers et al., 1997, 1998b
PND12	MS	24 h	PND13	–	↑	Rats (Wistar)	Vázquez et al., 1996
PND17	MS	24 h	PND18	–	↑	Rats (SD-LE hybrids)	Dent et al., 2000a
PND19	MS	24 h	PND20	–	↓	Rats (SD-LE hybrids)	Smith et al., 1997
PND1–14	MS	3 h	Adult	–^♂^	↑^♂^	Rats (LE)	Liu et al., 2000a
PND1–14	MS	3 h	Adult	–^♂^	↑^♂^	Rats (Wistar)	Veenema et al., 2006
PND1–14	ESD	4 h	Adult	–	–	Rats (Fisher)	Rüedi-Bettschen et al., 2006
PND1–14	ESD	4 h	Adult	–^♂^	–^♂^	Rats (Wistar)	Rüedi-Bettschen et al., 2005
PND1–21	ESD	4 h	Adult	–^♂^	–^♂^	Rats (Wistar)	Pryce et al., 2003
PND2–10	MS	6 h	Adult	–^♂^	–^♂^^*^	Rats (SD)	Rhees et al., 2001
PND2-13	MS	4 h	Adult	–	–^♂^/↓^♀^^$^	Rats (SD)	Chen et al., 2012
PND2–14	MS	3 h	Adult	– ^♂^	↑^♂^	Rats (LE)	Huot et al., 2004; Ladd et al., 2004; Plotsky et al., 2005; Lippmann et al., 2007
PND2–14	MS	3 h	Adult	–^♂^	↑^♂^	Rats (LE)	Ladd et al., 2005
PND2–14	MS	5 h	Adult	–^♀^	–^♀^	Rats (SD)	Rees et al., 2006
PND2–14	ESD	5 h	Adult	–^♀^	–^♀^	Rats (SD)	Rees et al., 2006
PND3	MS	24 h	Adult	–^♂^	–^♂^	Rats (Brown Norway)	Workel et al., 2001
PND3–15	MS	3 h	Adult	–	–	Rats (LE)	Slotten et al., 2006
PND5	MS	24 h	Adult	–^♂^	↑^♂^	Rats (LE)	Penke et al., 2001
PND14	MS	24 h	Adult	–^♂^	↑^♂^	Rats (LE)	Penke et al., 2001
PND3	MS	24 h	Elderly	–^♂^	–/↓^♂^^%^	Rats (Brown Norway)	Workel et al., 2001

If no sex is specified, results apply to both males and females. ↓ Indicates a significant decrease, ↑ a significant increase, and – no significant difference in ACTH plasma levels. Stressors are applied daily for the indicated period.

♂ Results apply to males;

♀ results apply to females;

@ increase only seen in lactating, not virgin females;

# applied on alternating days;

* peak response levels are unaffected but stress lasts significantly longer;

$ peak response levels are unaffected but the stress response is shorter-lasting;

& results differ between mouse strains tested;

% results are stressor-dependent;

∧ *cohort-differences; ESD, early social deprivation; GD, gestational day; LE, Long Evans rats; LN, limited nesting; MS, maternal separation; PND, postnatal day; SD, Sprague Dawley rats*.

#### Adrenals

PS (or prenatal corticosterone) generally increases corticosterone stress responses by elevating peak levels or increasing the total duration of the response (see Table 3), which both appear indicative of impaired negative feedback. Overall, these effects appear slightly stronger in PS females than males (Brunton and Russell, 2010; Table 3, Box 3). Basal corticosterone levels seem to be either increased or unaffected by PS (Table 3).

**Table 3:** **Overview of corticosterone plasma levels in prenatally and neonatally stressed animals as compared to non-stressed controls**.

**Period**	**Stress paradigm**	**Duration**	**Age at testing**	**Basal CORT**	**Stress-induced CORT**	**Species (strain)**	**References**
GD14–21	Restraint	3 × 45 min	PND3	–^♂^	↑^♂^	Rats (Wistar)	Henry et al., 1994
GD14–21	Restraint	3 × 45 min	PND21	–^♂^	↑^♂^	Rats (Wistar)	Henry et al., 1994
GD1/5^***^-21	Injection	daily	PND23	–	↑	Rats (SD)	Peters, 1982
GD1–7	Variable		Adult	–^♂^	↑^♂^	Mice (C57Bl/6:129)	Mueller and Bale, 2008
GD1–21	CBX	daily	Adult	↑^♂^	–^♂^	Wistar rats	Welberg et al., 2000
GD1–21	Hypoxia and/or restraint	4 h	Adult	↑^♂^		SD rats	Fan et al., 2009
GD1–21	Noise-light	3 × 4 h/week	Adult	–^♂^/↑^♀^	–^♂^/↑^♀^^*^	Rats (Sabra)	Weinstock et al., 1992
GD1–21	Noise-light	3 × 4 h/week	Adult	↑^♂^	↑^♂^	Rats (SD)	Weinstock et al., 1998
GD2–20	Foot shocks	Daily	Adult	–^♂^	↑^♂^	Rats (Wistar)	Sadler et al., 2011
GD4–10	Defeat or restraint^#^	45 or 60 min	Adult	–^♀^	↑^♀^^**^	Rats (Wistar)	Bosch et al., 2007
GD7–13	Variable		Adult	–^♂^	–^♂^	Rats (SD)	Koenig et al., 2005
GD9–20	Restraint	6 h	Adult	–^♂^	↑^♂^^*^	Mice (ICR)	Chung et al., 2005
GD11–18	Defeat	1 h	Adult	–^♀^	↑^♀^^**^	Rats (Wistar)	Bosch et al., 2007
GD11–18	Predator odor exposure	1 h	Adult	–	–^♂^/↑^♀^	Mice (C57BL/6)	St-Cyr and McGowan, 2015
GD14–21	Restraint	2 × 45 min	Adult	–^♂^	↑^♂^^*^	Rats (SD)	Vallée et al., 1996
GD14–21	Restraint	3 × 45 min	Adult	–^♂^	↑^♂^	Rats (Wistar)	Barbazanges et al., 1996
GD14–21	Restraint	3 × 45 min	Adult	–^♂^	↑^♂^^*^	Rats (Wistar)	Maccari et al., 1995
GD14–21	Restraint	3 × 45 min	Adult	–^♂^	↑^♂^^*^	Rats (Wistar)	Henry et al., 1994
GD14–21	Handling, novelty, injection	Daily	Adult	↑^♂^		Rats (SD)	Ward et al., 2000
GD14–22	Variable	Daily	Adult	–^♂^	↑^♂^^*^	Rats (SD)	Koenig et al., 2005
GD15–19	Restraint	20 min	Adult	–	–^♂^/↑^♀^	Rats (LE)	McCormick et al., 1995
GD15–19	Restraint	3 × 30 min	Adult	–^♂^/↑^♀^	–^♂^/↑^♀^^*^	Rats (Wistar-HAN)	Szuran et al., 2000
GD15–20	DEX	Daily	Adult	↑^♂^	–^♂^	Rats (Wistar)	Levitt et al., 1996
GD15–21	Restraint	60 min		–^♂^	↑^♂^	Rats (Wistar)	Hosseini-sharifabad and Hadinedoushan, 2007
GD15–21	Restraint	3 × 45 min	Adult	↑^♀^		Rats (Wistar)	Pérez-Laso et al., 2008
GD15–21	Restraint	3 × 45 min	Adult	–^♂^	↑^♂^^*^	Rats (SD)	Vallée et al., 1997
GD16–20	Defeat	10 min	Adult	–^♂^/ –/↑^♀^^∧^	↑	Rats (SD)	Brunton and Russell, 2010
PND1–14	MS	3 h	PND3	–^♂^	↑^♂^	Rats (SD)	Lajud et al., 2012
PND1–14	MS	3 h	PND6	–^♂^		Rats (SD)	Lajud et al., 2012
PND1–14	MS	3 h	PND9	–^♂^		Rats (SD)	Lajud et al., 2012
PND1–14	MS	3 h	PND12	–^♂^	↓^♂^	Rats (SD)	Lajud et al., 2012
PND2–9	LN		PND9	↑		Mice (C57BL/6)	Liao et al., 2014
PND2–9	LN		PND9	↑^♂^		Mice (C57Bl/6J)	Naninck et al., 2015
PND2–9	LN		PND9	↑		Mice (C57BL/6J)	Rice et al., 2008
PND2–9	LN		PND9	↑^♂^		Rats (SD)	Brunson et al., 2005
PND2–9	LN		PND9	↑		Rats (SD)	Avishai-Eliner et al., 2001
PND2–9	LN		PND9	–	↑^*^	Rats (SD-derived)	Gilles et al., 1996
PND2-9	LN		PND10	↓		Rats (Wistar)	Moussaoui et al., 2016
PND2–9	LN		PND10	–	↓	Rats (SD)	McLaughlin et al., 2016
PND2–9	LN		PND28	–^♂^	↓^♂^	Mice (C57Bl/6J)	Arp et al., 2016
PND2–9	MS	15 min	PND21	–		Rats (Wistar)	Moussaoui et al., 2017
PND2–10	LN		PND21	–^♂^/↑^♀^		Rats (Wistar)	Moussaoui et al., 2017
PND2–14	MS	5 h	PND30	–^♀^	–^♀^	Rats (SD)	Rees et al., 2006
PND2–14	ESD	5 h	PND30	–^♀^	↓^♀^^#^	Rats (SD)	Rees et al., 2006
PND3	MS	24 h	PND4	↑		Rats (Wistar)	Oomen et al., 2009
PND3	MS	24 h	PND4	–	↑	Rats (SD-LE hybrids)	van Oers et al., 1998b
PND3	MS	24 h	PND20	–	–	Rats (SD-LE hybrids)	van Oers et al., 1997, 1998b
PND4	MS	24 h	PND5	↑	↑	Mice (CD1 + C57BL/6J)	Daskalakis et al., 2014
PND5	MS	24 h	PND6	↑	↑	Rats (SD)	Avishai-Eliner et al., 1995
PND5	MS	24 h	PND6	–	↑^&^	Rats (SD-LE hybrids)	Dent et al., 2000a
PND5	ESD	24 h	PND6	↑	↑	Rats (SD)	Avishai-Eliner et al., 1995
PND6	MS	24 h	PND7	–	↑	Rats (Wistar)	Vázquez et al., 1996
PND7	MS	24 h	PND20	–	–	Rats (SD-LE hybrids)	van Oers et al., 1997
PND8	MS	24 h	PND9	↑		Rats (SD)	Eghbal-Ahmadi et al., 1997
PND8	MS	24 h	PND9	↑	↑	Rats (SD)	Avishai-Eliner et al., 1995
PND8	ESD	24 h	PND9	↑	↑	Rats (SD)	Avishai-Eliner et al., 1995
PND9	MS	24 h	PND10	–	↑	Rats (Wistar)	Vázquez et al., 1996
PND11	MS	24 h	PND12	↑	↑	Rats (SD-LE hybrids)	Smith et al., 1997; van Oers et al., 1998a,b
PND11	MS	24 h	PND12	↑	↑	Rats (SD-LE hybrids)	Dent et al., 2000a
PND11	MS	24 h	PND16	–	↓^$^	Rats (SD-LE hybrids)	van Oers et al., 1997
PND11	MS	24 h	PND20	–	↓^$^	Rats (SD-LE hybrids)	van Oers et al., 1997
PND11	MS	24 h	PND20	–	–	Rats (SD-LE hybrids)	van Oers et al., 1998b
PND12	MS	24 h	PND13	–	↑	Rats (Wistar)	Vázquez et al., 1996
PND17	MS	24 h	PND18	–	↑	Rats (SD-LE hybrids)	Dent et al., 2000a
PND19	MS	24 h	PND20	–	–	Rats (SD-LE hybrids)	Smith et al., 1997
PND2–6	ESD	5 h	Adolescent	↑^♂^	↑^♂^	Rats (SD)	Biagini et al., 1998
PND1–10	MS	3 h	Adult	↑^♂^	↑^♂^	Mice (C57Bl/6N)	Murgatroyd et al., 2009
PND1–10	MS	3 h	Adult	↑^♂^		Mice (C57Bl/6N)	Wu et al., 2014
PND1–14	MS	3 h	Adult	–^♂^		Rats (SD)	Mirescu et al., 2004
PND1–14	MS	3 h	Adult	–^♂^	–^♂^	Rats (Wistar)	Veenema et al., 2006
PND1–14	MS	3 h	Adult	↑^♂^	↑^♂^	Rats (SD)	Lajud et al., 2012
PND1–14	ESD	4 h	Adult	–	↓^♂^*/*–^♀^	Rats (Fisher)	Rüedi-Bettschen et al., 2006
PND1–14	ESD	4 h	Adult	–^♂^	–^♂^	Rats (Wistar)	Rüedi-Bettschen et al., 2005
PND1–15	ESD	4 h	Adult	–^♂^		Rats (Wistar)	Marmendal et al., 2006
PND1–21	ESD	3 h	Adult	–^♂^	↓^♂^	Rats (SD)	Zhang et al., 2014
PND1–21	ESD	4 h	Adult	–	–^♂^/↓^♀^	Rats (Wistar)	Pryce et al., 2003
PND2–9	LN		Adult	–^♂^		Rats (SD)	Brunson et al., 2005
PND2–9	LN		Adult	–		Mice (C57Bl/6J)	Naninck et al., 2015
PND2–9	LN		Adult	↑^♂^		Mice (C57Bl/6J)	Rice et al., 2008
PND2–9	LN		Adult	–^♂^	–^♂^	129S2/Sv × C57Bl/6J mice	Wang et al., 2012
PND2–9	LN		Adult	↑^♂^/↓^♀^		Mice (C57Bl/6J)	Arp et al., 2016
PND2–10	MS	6 h	Adult	–	–^♂^/↑^♀^^*^	Rats (SD)	Rhees et al., 2001
PND2–13	MS	4 h	Adult	–^♂^/↑^♀^	↑^&^	Rats (SD)	Chen et al., 2012
PND2–14	MS	3 h	Adult	–^♂^	–^♂^	Rats (LE)	Huot et al., 2004; Ladd et al., 2004
PND2–14	MS	3 h	Adult	–^♂^	↑^♂^	Rats (LE)	Plotsky and Meaney, 1993; Ladd et al., 2005; Plotsky et al., 2005; Lippmann et al., 2007
PND2–14	MS	5 h	Adult	–^♀^	–^♀^	Rats (SD)	Rees et al., 2006
PND2–14	ESD	5 h	Adult	↑^♀^	↓^♀^	Rats (SD)	Rees et al., 2006
PND2–21	MS	3 h	Adult	↑^♀^		Rats (Wistar)	Aisa et al., 2008
PND3	MS	24 h	Adult	–/↑^♂^^¥^	–/↑/↓^♂^^¥^	Rats (Brown Norway)	Workel et al., 2001
PND3–15	MS	3 h	Adult	↓	–	Rats (LE)	Slotten et al., 2006
PND4	MS	24 h	Adult	–^♂^	↑/–^♂^^¥^	Rats (Wistar)	Lehmann et al., 2002
PND5	MS	24 h	Adult	–^♂^	↑^♂^	Rats (LE)	Penke et al., 2001
PND9	MS	24 h	Adult	–^♂^	↑/–^♂^^¥^	Rats (Wistar)	Lehmann et al., 2002
PND14	MS	24 h	Adult	↓^♂^	↑^♂^^*^	Rats (LE)	Penke et al., 2001
PND18	MS	24 h	Adult	–^♂^	↑/–^♂^^¥^	Rats (Wistar)	Lehmann et al., 2002

*If no sex is specified, results apply to both males and females. ↓ Indicates a significant decrease, ↑ a significant increase, and – no significant difference in corticosterone plasma levels. Stressors are applied daily for the indicated period unless specified otherwise*.

♂ Results apply to males;

♀ results apply to females;

* peak response levels are unaffected but the stress response lasted significantly longer;

** increased levels only observed in lactating rats, not in virgins;

*** exact start of PS not stated;

# peak response levels are unaffected but corticosterone levels rise significantly slower;

$ peak response levels are unaffected but the stress response is shorter-lasting;

& peak response levels are unaffected but are reached sooner;

¥ age-dependent effects;

∧ *cohort-differences; CORT, corticosterone; DEX, dexamethasone, ESD, early social deprivation; GD, gestational day; LE, Long Evans rats; LN, limited nesting; MS, maternal separation; PND, postnatal day; SD, Sprague Dawley rats*.

Effects of neonatal stress on adrenal function are again stressor-specific, and depending on the developmental period affected and the age at which they are assessed (Table 3). The LN model generally induces elevated basal corticosterone levels during the SHRP, which can be prevented by either GR- (in females) or CRHR1- blockage (in both sexes; Liao et al., 2014). However, these levels (as well as adrenal weight) seem to have normalized in adulthood (Naninck et al., 2015), although sex-specific effects might exist; whereas some studies observed increased corticosterone levels and adrenal weight in LN males (Rice et al., 2008; Arp et al., 2016), decreased levels were observed in females (Arp et al., 2016). Corticosterone stress responses have been shown to be either prolonged (Gilles et al., 1996), unaffected (Wang et al., 2012), or reduced (McLaughlin et al., 2016) as a consequence of LN. The effects of 24 h MS seem to be strongly age-dependent as well. MS applied during the SHRP increases both basal and stress-induced corticosterone levels observed during the SHRP (Table 3), without affecting basal corticosterone levels and exerting only minimal effect on stress-induced corticosterone levels when assessed later during infancy. Increased basal levels, but reduced stress-response levels are observed in 3 month-old rats (Workel et al., 2001), whereas in 5 and 12 month-olds basal levels are unaltered, but stress-response levels increased as a consequence of MS (Workel et al., 2001; Lehmann et al., 2002). In elderly rats (20 months), basal and stress-induced levels are again unaffected (Lehmann et al., 2002), whereas stress-induced levels are reduced at senescent age (Workel et al., 2001). Similarly, multiple-day MS does not seem to induce any consistent alterations in basal corticosterone levels either during the SHRP or adulthood (Table 3). Corticosterone stress responses are however typically increased as a consequence of this repeated stressor. Lastly, ESD has been shown to increase basal and stress-induced corticosterone levels during the SHRP (24 h ESD) and in adolescence (PND45) (multiple-day ESD), though in juveniles (PND30) multiple-day ESD was not found to affect basal corticosterone levels, and slowed down stress-induced release. In adulthood, basal corticosterone levels generally are similar to levels observed in non-stressed controls. Interestingly and in contrast to MS, ESD seems to induce *reduced* stress-response corticosterone levels in adulthood (Table 3).

These ELS-induced alterations in corticosterone plasma levels could obviously be caused by the earlier mentioned alterations in CRH and ACTH release, but could also be attributed to abnormal function of the adrenal gland itself, as increases in adrenal weight and cortex-to-medulla ratio have been reported as a consequence of PS (Ward et al., 2000; Fan et al., 2009; Liaudat et al., 2015). However, the frequent inconsistencies in findings emphasize the extremely complex modulatory effects ELS exerts on the HPA-axis, depending on the precise developmental stage affected, the exact stressor used (its frequency, duration, etc.), age of testing, sex of the offspring, and also the genetic background of the animals. Structured assessment of these effects is absolutely necessary to increase understanding of the underlying mechanisms of aberrant corticosteroid signaling later in life.

### Developmental effects of ELS on HPA-axis modulators

#### Amygdala

The amygdala plays a prominent role in the behavioral fear response and the regulation of emotional processing (Akirav and Maroun, 2007). CRH-expressing cells (first detected at PND6, after which they gradually increase with age; Vazquez et al., 2006) are quite abundant, particularly in the CeA, a major output site which projects to the hypothalamus (LeDoux et al., 1988; Gray et al., 1989). Activation of GRs expressed on CeA CRH-neurons increases local CRH mRNA expression (Makino et al., 1994), which directly contributes to a state of fear (Kolber et al., 2008). These CRH-containing neurons project through the bed nucleus in the stria terminalis to the PVN, and are believed to stimulate the HPA-axis and induce anxiety-like behavior (Feldman et al., 1994; Brunson et al., 2001a). Simultaneously, CRH released by the PVN activates the amygdala to increase anxiety (Schulkin, 2006), forming a potent feed-forward loop in stress signaling.

The amygdala develops both pre- and postnatally. It emerges during the third week of gestation, but matures prominently throughout infancy and adolescence (Berdel et al., 1997), changing neuronal morphology (Ryan et al., 2016), intrinsic membrane properties, action potential kinetics, and the synaptic and voltage-gated currents (Ehrlich et al., 2012, 2013). From PND7–21 in rats, regional soma volume doubles, spine density increases nearly five-fold, whereas dendritic arbors expand throughout the first postnatal month (Ryan et al., 2016). Neuronal density however reduces postnatally (Berdel et al., 1997).

PS influences the developmental trajectories of the rats' amygdalar subnuclei; the BLA, CeA, and lateral (LA) amygdala. Development of these regions was shown to be temporarily impeded by PS, with at offspring displaying significant reductions in regional volume and neuronal and glial number at PND25, which normalized at PND60 (Kraszpulski et al., 2006). In line with this, increased apoptosis was observed in the amygdala of pups (at PND7) as a consequence of prenatal corticosteroid treatment (Zuloaga et al., 2011), and an altered balance in subunit expression of glutamatergic and GABAergic receptors was observed at PND14–22 following prenatal restraint (Laloux et al., 2012). However, in another study the same stressor *increased* the volume and neuronal and glial number of the LA—the subregion serving as the site of signal-input from the sensory processing systems (LeDoux, 1994)—at PND80–120, without affecting the other subregions (Salm et al., 2004), suggesting age-dependent effects. Cell proliferation in the infant amygdala showed a non-significant reduction as a consequence of PS (Kawamura et al., 2006), whereas electrophysiological recordings from BLA excitatory principal neurons revealed a hyperpolarized resting membrane potential, larger action potential after-hyperpolarizations and H–currents in PS rat offspring compared to controls, reducing neuronal excitability throughout development from infancy into young adulthood (PND60; Ehrlich and Rainnie, 2015).

Whereas PS thus appears to transiently impede amygdala development, stress applied to the neonate seems to hasten amygdala maturation. Typically, the amygdala is not activated by aversive experiences shortly after birth (until PND8) and pups show attenuated learning of fear (and an approach response to aversively conditioned stimuli; Sullivan et al., 2000), which seems to be crucial for forming dam-pup attachment (Sullivan and Holman, 2010). Neonatal stress however accelerates the development of an aversive response and precocious activation of the amygdala, with pups expressing aversive learning and significant corticosterone stress responses at PND8 when reared in the LN model (Moriceau et al., 2006, 2009). This acceleration seems to be mediated by increased corticosteroid exposure, as corticosteroid infusion in the amygdala mimics the effects (Moriceau et al., 2006) and the administration of a corticosteroid receptor antagonist prevents them (Moriceau et al., 2009). In fact, suppressed aversion learning may be another reason for the SHRP, reducing corticosterone exposure to allow proper dam-pup attachment to occur. Neonatal stress also leads to longer fear retention (Callaghan and Richardson, 2012) and precocious expression of the mature form of extinction learning (Callaghan and Richardson, 2011; Cowan et al., 2013); all suggesting a (premature) acceleration in amygdala development of the stressed neonate. Amygdalar connectivity is affected by this early “maturation,” as myelination is expedited due to ELS (Ono et al., 2008). Potentially, this strengthening of early connections (e.g., those to the thalamus and nucleus accumbens) comes at the expense of the connections that form later in development, including those to the frontal cortex (Bouwmeester et al., 2002). Support for this idea comes from the preclinical observation of aberrant functional amygdala-frontal cortex connectivity in adolescents and adults that experienced childhood adversity (Birn et al., 2014; Fan et al., 2014; Lee et al., 2015). Alternatively, these changes in connectivity could derive from the precocious closing of a critical period of plasticity through neonatal stress. Closure of such critical periods has been shown to coincide with the emergence of perineuronal nets on parvalbumin interneurons (Pizzorusso et al., 2002; Hensch, 2005; Dityatev et al., 2007; Nowicka et al., 2009), stabilizing synapses. MS was shown to increase the number of parvalbumin neurons in the periadolescent LA (Giachino et al., 2007; Seidel et al., 2008), but the effects of stress on the perineuronal nets still have to be characterized. Gross amygdala morphology however does not seem to be affected by MS (Krugers et al., 2012).

Functionally, the adult amygdala seems to be come “overactive” as a consequence of ELS. Assessment of regional cerebral blood flow (CBF) by autoradiography revealed an increased cerebral activation of the amygdala in adult (~PND100) PS offspring to a fear-conditioned stimulus (Laviola et al., 2004), which was accompanied by heightened fear responsivity (i.e., freezing behavior; Sadler et al., 2011). However, also increased amygdala and fear responsivity to the tone was observed without any prior conditioning, suggesting general amygdala hyperactivity and increased anxiety in the PS animals (Sadler et al., 2011). In line with elevated amygdala activity, PS or exposure to elevated corticosteroid levels during gestation was shown to increase amygdala's basal CRH mRNA levels (Welberg et al., 2001; Brunton and Russell, 2010), as well as local CRH release in adult animals (Cratty et al., 1995). MS was found to leave local basal CRH mRNA expression unaffected (Bravo et al., 2011), but ESD, a more severe stressor, was shown to increase stress-induced levels (Barna et al., 2003). These findings may be related to local changes in the inhibition of CRH-induced activation as regulated by local GABAergic signaling. GABAa receptor binding was found to be reduced in the CeA and BLA as a consequence of MS (Caldji et al., 2000), joined by an increase in α2/α3 and decrease in α1 subunit mRNA expression; a profile associated with decreased GABA binding (Wilson, 1996). Moreover, these findings might relate to the altered methylation patterns of the *Crh* promoter as a consequence of ELS, which correlated with CRH mRNA levels in the central amygdala in a learned helplessness paradigm, but their direction depends on the genetic background of the animal (van der Doelen et al., 2015). The influence of ELS on local CRHR expression seems to be age-, sex-, and subregion-specific. PS was found to elevate CRHR1 mRNA expression in the CeA and BLA of males, and in the MeA of females (Brunton et al., 2011), whereas CRHR2 mRNA expression was not affected in the BLA and MeA, but reduced in the basomedial amygdala of males and increased in females (Brunton et al., 2011). MS was found to increase CRHR1 mRNA expression in the MeA during infancy, and decrease CRHR1 and CRHR2 mRNA levels in the CeA (Vázquez et al., 2003). However, in adulthood CeA and BLA CRHR1 mRNA expression levels are actually elevated in MS offspring, and BLA CRHR2 mRNA expression is reduced (Bravo et al., 2011). Importantly, no effects of neonatal stress on CRHR1/2 mRNA expression levels in adulthood are observed when the amygdala is considered as a whole (O'Malley et al., 2011), emphasizing the relevance of studying subregion-specific expression profiles. MS also affects the rather immediate alterations in receptor expression typically observed following acute stress. It attenuates the typical decrease in CRHR1 mRNA expression and raises CRHR2 mRNA levels in response to an acute psychological stressor (O'Malley et al., 2011). As CRHR1 activation by CRH in the amygdala typically serves an activating, anxiogenic role (Dunn and Berridge, 1990; Henckens et al., 2016), elevated expression levels match the overall increase in anxiety-like behavior of ELS animals. In line with this, injection of a CRHR antagonist abolished the increased fear and sensitivity to the environment of the PS offspring (Ward et al., 2000).

ELS also affects corticosteroid signaling in the amygdala. PS was found to increase CeA GR mRNA levels (Brunton and Russell, 2010) and overall GR-binding (McCormick et al., 1995). These effects might be mediated by elevated corticosteroid exposure of the fetus, as GR (but not MR) mRNA levels in the BLA, CeA, and MeA were found to be increased by the inhibition of 11β-HSD2 (Welberg et al., 2000), and BLA MR and GR mRNA expression were increased as a consequence of dexamethasone administration during pregnancy (Welberg et al., 2001). Remarkably, GR expression in the amygdala was found to be *reduced* in MS offspring, although this effect might be strain-specific. MS reduced amygdala basal GR mRNA expression during the SHRP in C57Bl/6J mice, but not in CD1s (Daskalakis et al., 2014), and this decrease remained present until adulthood (Arnett et al., 2015). Despite the fact that neonatal stress typically induces an anxiogenic phenotype (Huot et al., 2002; Kalinichev et al., 2002; Daniels et al., 2004; Rees et al., 2006; Aisa et al., 2007; Trujillo et al., 2016), this apparent decrease in GR expression was associated with reduced anxiety of the ELS animals compared to controls, which was normalized by lentiviral-mediated restoration of GR levels (Arnett et al., 2015).

#### Hippocampus

The hippocampus, best-known for its role in spatial learning and memory (Block and Schwarz, 1997), plays an important inhibitory role in the regulation of the HPA-axis by its direct and indirect polysynaptic connections to the PVN. Electric stimulation of hippocampal subfields [CA3, dentate gyrus (DG), and subiculum] reduces corticosteroid release (Dunn and Orr, 1984), whereas hippocampal lesions and those of the ventral subiculum increase CRH mRNA levels in the PVN (Herman et al., 1989), and prolong the corticosterone stress response (Herman et al., 1995), respectively. This feedback seems to be relayed to the hypothalamus by indirect projections through the bed nucleus stria terminalis (Herman et al., 2003). Because of its high local GR/MR expression levels, moderate CRHR1/2 levels, and local CRH-expression, the hippocampus is however highly sensitive to the influences of stress (de Kloet et al., 1990; Maras and Baram, 2012). The first 2 postnatal weeks comprise a crucial period in hippocampal maturation (Frotscher and Seress, 2007), as this is when the hippocampal commissural/associational (C/A) pathways establish their synaptic connections on CA3 pyramidal cell dendrites (Bayer, 1980). Disruption of this process can only be partially restored beyond the third postnatal week (Gall and Lynch, 1978), making that stress experienced during this period can profoundly affect hippocampal structure and function.

ELS has been shown to slow the acquisition of spatial learning and/or impair memory under both moderately stressful and relatively stress-free conditions (Lemaire et al., 2000; Huot et al., 2002; Brunson et al., 2005; Ishiwata et al., 2005; Yang et al., 2006b; Aisa et al., 2007; Kosten et al., 2007; Rice et al., 2008; Ivy et al., 2010; Hulshof et al., 2011). In one of these studies, PS-induced learning deficits were associated with a reduction in spine density of pyramidal neuron dendrites in the hippocampal CA3 region (Ishiwata et al., 2005). Other studies confirmed this PS-reduced spine density not only in the CA3, but also the CA1 subregion of the hippocampus (Martínez-Téllez et al., 2009). Besides, PS reduced dendritic length and branching of CA3, but not CA1, neurons (Hosseini-sharifabad and Hadinedoushan, 2007). Similar reductions in spine density of CA1 neurons were observed as a consequence of ESD and LN, which was, in contrast to the case of PS, joined by CA1 dendritic atrophy (Ivy et al., 2010; Monroy et al., 2010). Moreover, LN was found to reduce apical dendritic length and neuronal complexity in CA3 neurons in infants (Liao et al., 2014). Whereas MS decreased the density of mossy fibers in the stratum oriens (Huot et al., 2002), no changes in apical dendritic length and neuronal complexity have been found in the DG (Oomen et al., 2011).

These structural alterations affect local synaptic plasticity; PS impairs long-term potentiation (LTP) in the CA1 (which is associated with a decreased expression and impaired interaction of the NR1 and NR2B subunits of the NMDA receptor in hippocampal synapses; Son et al., 2006), whereas long-term depression (LTD) is facilitated. Furthermore, PS was shown to enhance the effects of acute stress on impairing hippocampal LTP and facilitating LTD (Yang et al., 2006a). Cross-fostering the neonate offspring with control mothers did not change these effects on hippocampal LTP and LTD, implicating they resulted directly from the prenatal manipulation and not altered maternal care (see Box 2; Yang et al., 2006a). However, environmental enrichment after weaning restored plasticity in PS animals, as well as the associated impairments in spatial memory (Yang et al., 2007), emphasizing the impact of the neonate's environment on PS effects. Not surprisingly, disturbed LTP in the CA1, CA3, and DG is also observed as a consequence of stress in the neonate (by both LN and MS; Brunson et al., 2005; Cui et al., 2006; Ivy et al., 2010; Batalha et al., 2013; Cao et al., 2014; Xiong et al., 2014). However, these perturbations are not always found and may depend on the developmental stage affected by stress (Gruss et al., 2008), the sex of the animal (Oomen et al., 2011), and the age of testing (Brunson et al., 2005). Moreover, they might depend on the exact ELS model implemented, since ESD has been found to enhance DG LTP induction and duration in juvenile (Kehoe et al., 1995; Bronzino et al., 1996) and adult (Kehoe and Bronzino, 1999) offspring. Potentially in line with this ESD-boosted hippocampal LTP is the observation that neonatal isolation accelerates the developmental switch in the signaling cascades for local LTP induction (Huang et al., 2005). However, ESD was also shown to prevent acute stress-induced potentiation of LTP in the DG (Wang H. et al., 2013). Future studies should further elucidate the critical dependables in the modulation of the effects of ELS on hippocampal plasticity.

Although several studies have attributed these effects to elevated corticosteroid exposure of the hippocampus (Brunson et al., 2005), suppressing dendritic growth and branching (Alfarez et al., 2009; Liston and Gan, 2011), the presence of both elevated levels of CRH and CRHR1 (with CRHR1 mRNA expression detected at ~300–600% of adult levels at PND6; Avishai-Eliner et al., 1996) during early developmental stages points toward their critical role in development (and thereby particular sensitivity of the brain to their dysregulation). Hippocampal CRH-immunoreactive neurons are already detected at PND1 (Yan et al., 1998; Chen et al., 2001) and numbers increase to peak levels at PND18, after which levels reduce to those observed in adulthood (Chen et al., 2001). Interestingly, at this initial stage of development, hippocampal CRH mRNA is not only detected in basket- and chandelier-type GABAergic interneurons (Yan et al., 1998; Chen et al., 2001) synapsing on somata of hippocampal pyramidal neurons, but also a second population of CRH-expressing neurons is present, possessing the morphology of hippocampal Cajal-Retzius cells. These non-GABAergic neurons disappear by the end of the second postnatal week (Chen et al., 2001), but emphasize the potential modulatory role CRH can have during early development. CRH is tonically released in the hippocampus, as becomes apparent from the abnormal dendritic structure (i.e., hypertrophy), spine morphology, and impaired synaptic potentiation and spatial learning observed when CRHR1s are chronically blocked (Chen et al., 2004) and in mice lacking CRHR1 (Contarino et al., 1999; Schierloh et al., 2007; Wang et al., 2011). However, the balance seems to be critical. CRH applied to slice cultures was shown to reduce spine density (Chen et al., 2008) and induce dendritic atrophy (Lin and Koleske, 2010), whereas CRH administration into the hippocampus recapitulated the learning and memory problems associated with ELS (Brunson et al., 2001b). Importantly, all these effects are observed when corticosteroid levels are maintained at basal levels. Additionally, both CRH mRNA and protein levels are generally upregulated in ELS animals (Wang et al., 2014), the number of CRH expressing interneurons in the CA1 and CA3 is increased (Ivy et al., 2010), and blockage of CRHR1 prevents dendritic atrophy and LTP attenuation, as well as the impairment in memory performance observed in neonatally stressed animals (Ivy et al., 2010). Therefore, elevated CRHR1-activation has been suggested to mediate the ELS effects on hippocampal function (Maras and Baram, 2012); a hypothesis that was further corroborated by the observation that mice lacking CRHR1 are resistant to the detrimental effects of ELS on hippocampal function (Wang et al., 2011).

ELS also affects neurogenesis in the DG, one of the brain's only sites that displays neurogenesis well into adulthood (Drew et al., 2013). Reductions in adult neurogenesis and cell proliferation are observed as a consequence of PS (Lemaire et al., 2000; Morley-Fletcher et al., 2011; Belnoue et al., 2013), with the severity of the reduction depending on the severity of the PS paradigm and gestational stage affected (Mandyam et al., 2008; Madhyastha et al., 2013), with stress later in pregnancy inducing stronger effects. As the DG for the larger part develops postnatally (Altman and Bayer, 1990a,b), this structure may be particularly sensitive to stress during the first weeks of life. In line with this, it was shown that neonatal stress strongly affects DG neurogenesis in a sex-, age-, and possibly species-specific manner. When assessed at the end of the SHRP, neurogenesis was found to be reduced in rats as a consequence of MS (Lajud et al., 2012), but increased in mice exposed to LN (Naninck et al., 2015). At the age of weaning, sex-specific effects were observed following MS, with male rats showing increased neurogenesis, whereas female rats displayed decreased levels (Oomen et al., 2009). Sex-specific effects of ELS were also observed in adulthood, but in an opposite direction; adult neurogenesis was reduced in MS and LN males (Oomen et al., 2010; Lajud et al., 2012; Naninck et al., 2015), whereas no effects were found in females (Oomen et al., 2011; Naninck et al., 2015). For cell death, conflicting results have been found, ranging from unaffected levels in both sexes (Lemaire et al., 2000; Mandyam et al., 2008), to increased levels in PS males (Mandyam et al., 2008).

Potentially related to these effects on neurogenesis and cell survival, volume reductions have been observed in the DG as a consequence of LN (Naninck et al., 2015), but not MS (Huot et al., 2002). ELS is also reported to locally decrease neuron and glia cell numbers (Leventopoulos et al., 2007; Fabricius et al., 2008; Oomen et al., 2011). Other hippocampal regions were not found to be reduced in volume by ELS (Fabricius et al., 2008; Hui et al., 2011; Zalosnik et al., 2014). Cell proliferation seems to be particularly affected in the caudal/ventral part of the DG (Oomen et al., 2010; Hulshof et al., 2011), implying altered hippocampal contribution to emotional behaviors (Bannerman et al., 2004; Fanselow and Dong, 2010) as a consequence of ELS. Alterations in expression levels of the neurotrophic factor BDNF, which stimulates the survival of newborn cells and is involved in cell proliferation, might be mediating these effects on cell proliferation. Hippocampal BDNF levels in female adult offspring were found to be reduced as a consequence of PS, which was related to a decreased DNA methylation in *bdnf* exon IV. No such effects were however observed in the male offspring (St-Cyr and McGowan, 2015) and another study even reported on increased BDNF levels in PS males (Zuena et al., 2008). Reports on the effects of neonatal stress on BDNF are conflicting, as both increased (Roceri et al., 2004) and decreased BDNF mRNA expression (Kuma et al., 2004) have been observed in MS-exposed infants, and either similar BDNF mRNA (Roceri et al., 2004; Greisen et al., 2005) accompanied by increased BDNF protein levels (Greisen et al., 2005), decreased BDNF mRNA (Aisa et al., 2009), or increased BDNF mRNA levels (Kuma et al., 2004) have been observed in MS adults. Differences in duration and developmental phase affected by the MS paradigm might be responsible for these inconsistencies, although differences in rat strain might contribute as well.

Interestingly, although adult MS animals mostly show normal basal levels of corticosterone (see Table 3), depleting corticosterone (by adrenalectomy) can reverse this suppression of cell proliferation and neurogenesis, implicating inhibited cellular plasticity due to hypersensitivity to corticosterone signaling in the hippocampus (Mirescu et al., 2004). This abnormal sensitivity to corticosterone might be mediated by altered corticosteroid receptor expression or MR/GR balance as a consequence of ELS. PS has been found to decrease hippocampal MR mRNA levels, density, and binding capacity (Henry et al., 1994; Maccari et al., 1995; Koehl et al., 1999; Van Waes et al., 2006; Brunton and Russell, 2010), which could relate to the increased basal CRH levels in the PVN. Moreover, PS was shown to reduce hippocampal GR levels (Henry et al., 1994; Barbazanges et al., 1996; Koehl et al., 1999; Szuran et al., 2000; Chung et al., 2005; Van Waes et al., 2006; Mueller and Bale, 2008; Green et al., 2011; Bingham et al., 2013), attenuating its negative feedback on the HPA-axis, potentially explaining the stronger and prolonged corticosterone responses in PS animals (Chung et al., 2005; Koenig et al., 2005). These effects seemed to be mediated by increased prenatal corticosteroid exposure of the pups, as they were prevented by adrenalectomy in the mothers and reinstated by corticosterone injection in adrenalectomized dams (Barbazanges et al., 1996). MS during the SHRP induced an immediate decrease in CA1 MR (but not GR) mRNA expression in rat pups (Vázquez et al., 1996), whereas MS toward the end of the SHRP induced an immediate downregulation of both CA1 MR and GR mRNA expression (van Oers et al., 1998a). No effects were observed in the other hippocampal subregions in these studies. In adulthood, mixed effects of MS on hippocampal MR mRNA expression are found, with levels found to be either increased in all hippocampal subregions (Ladd et al., 2004), in the DG only (Workel et al., 2001), or unaffected (Ladd et al., 2005; Batalha et al., 2013). GR protein levels are however univocally downregulated in the hippocampus in neonatally stressed adults (Weaver et al., 2004; Aisa et al., 2007, 2008; Batalha et al., 2013; Arnett et al., 2015) although these effects not always translate to the mRNA level (Ladd et al., 2004, 2005; Brunson et al., 2005). Moreover, these effects may be sex-specific and occur only upon repeated stress exposures, as downregulation in GR and MR expression were observed in males, but upregulation of GR was observed in females as a consequence of 24 h MS (Sutanto et al., 1996). Overall, these alterations might result in an increased MR/GR ratio in the hippocampus (Ladd et al., 2004), which may result in an amplified initial stress reaction by increased activation of the membrane MR in a feed-forward fashion, and an impaired containment of this response by reduced membrane and genomic GR-mediated negative feedback (Oitzl et al., 2010).

Studies have recently focused on the putative association between DNA methylation at the GR gene (*NR3C1*) and ELS, mediating this reduction in GR expression (in males at least). This line of work started with the discovery by Weaver and colleagues that differential levels of maternal care critically modulated methylation levels of the GR promoter exon 1_7_, influencing local transcription factor (NGF1-A) binding, histone acetylation, and ultimately hippocampal GR expression and corticosterone responding in the offspring (Weaver et al., 2004). These differences emerged over the first week of life, were reversed by cross-fostering, and persisted into adulthood. Moreover, they were prevented by the central infusion of a histone deacetylase inhibitor, suggesting a causal relation among epigenomic state, GR expression and the maternal effect on stress responses in the offspring. These findings were replicated in a study in human suicide victims with a history of childhood abuse; the hippocampi of early life abuse victims were characterized by decreased GR mRNA levels, GR transcripts of the GR 1F-splice variant, as well as increased methylation of the *NR3C1* promoter (McGowan et al., 2009). Another recent study replicated this finding of enhanced DNA methylation at this splice variant and additionally identified altered DNA methylation in other splice variants of the GR promoter (Labonte et al., 2012). Moreover, it showed that this epigenetic response to ELS is brain region-specific, not occurring in the anterior cingulate. Studies like this, as well as the observation that epigenetic mechanisms critically contribute to conferring cell-type identity during development and cell division, suggest that the impact of environmental factors on epigenetic marks is likely to be to some extent cell-type specific, emphasizing the relevance of limiting analysis to appropriate tissues of interest instead of mere analyses of leukocytes (please see McGowan, 2013 for an extensive review on this issue). Nevertheless, these initial human data translate rodent findings to humans, suggesting a common effect of early life environment on the epigenetic regulation of hippocampal GR expression.

#### Prefrontal cortex

The PFC is key to stress coping and emotion regulation (Arnsten, 2009) through its inhibitory connections to both the amygdala (Banks et al., 2007) and the PVN, where it inhibits CRH release (Radley et al., 2006). It represses the HPA-axis predominantly through inhibitory projections from the infralimbic (IL), prelimbic (PL), and anterior cingulate cortex (ACC) that target HPA-axis neurons directly or indirectly (Heidbreder and Groenewegen, 2003), although the exact functional implications for the HPA-axis seem to be subregion-specific (Radley et al., 2006). It represents the functionally most advanced area of the brain with the longest period of maturation. This prolonged development allows for the acquisition of complex cognitive abilities through experience, but also makes it susceptible to factors that can lead to abnormal functioning, which is often manifested in neuropsychiatric disorders (Schubert et al., 2015). Its development starts prenatally with the proliferation and migration of neurons, growth of dendrites, the formation of neural micro- and macro-circuits through efferent/afferent axonal projections, but continues after birth with the initial overproduction of neurons and their connection being fine-tuned by reducing synaptic contacts (e.g., by the pruning and cell death of unused connections; Kolb et al., 2012) and neuronal density steered by experience.

ELS typically impairs PFC function in adulthood, as is exemplified by increased impulsivity (Gondré-Lewis et al., 2016), deficits in extradimensional shifts of attention (Mehta and Schmauss, 2011), and impaired working, short-term, and long-term memory (Gué et al., 2004; Markham et al., 2010; Negrón-Oyarzo et al., 2015; Alteba et al., 2016). In line with this, PS has been shown to impair prefrontal LTP, which was accompanied by an increase in the mean frequency of spontaneous excitatory postsynaptic currents (sEPSCs) in layer II/III pyramidal neurons (Sowa et al., 2015). Similar results have been observed in rats following chronic corticosterone treatment (Bartosz et al., 2011), suggesting a role for glucocorticoids in this impaired LTP. MS has been shown to result in LTP impairment in the IL layer II/III-layer V (Xiong et al., 2014), and ELS to impair extinction retrieval of context-dependent fear memories by preventing the synaptic potentiation of hippocampal-PL cortex neural pathway, which displayed synaptic inhibition rather than potentiation (Judo et al., 2010). Another study into the effects of PS confirmed this aberrant hippocampal-PFC functional connectivity as the temporal coupling between neuronal discharge in the medial PFC (mPFC) and hippocampal sharp-wave ripples was decreased by PS (Negrón-Oyarzo et al., 2015). In line with this, decreased regional CBF was elicited in the dorsal mid-cingulate and posterior cingulate cortex in MS rats in response to a conditioned tone compared to controls (Sadler et al., 2011).

These functional changes in the frontal cortex might be mediated by structural alterations caused by ELS. Comprehensive insight into the modulatory role of ELS on PFC neural morphology is derived from a series of experiments in a precocious rodent, the degu. In contrast to classical laboratory rats and mice, degus (like human babies) are born with relatively mature sensory systems and can thus perceive and more elaborately interact with their early life environment, making them a very suitable model to study the impact of neonatal stress (Bock and Braun, 2011; Braun and Bock, 2011). Brief MS increases corticosterone levels (Gruss et al., 2006) and both MS and ESD downregulate PFC activity during the separation period (Bock et al., 2012). Repeated separation has been shown to increase spine density in the basal dendrites of layer III dorsal ACC neurons in adolescent animals when compared to nonstressed controls (Helmeke et al., 2001). This finding could potentially be explained by either delayed or permanently impaired synaptic pruning during PFC development. The effect of this increased excitatory spine density may even be exaggerated by a decrease of inhibitory shaft synapses on the neurons by stress (Ovtscharoff and Braun, 2001), inducing a dysbalance of PFC synaptic input and neuronal output. Besides altering synaptic contacts, neonatal stress was shown to also affect the number and type of inhibitory interneurons in the ACC (Helmeke et al., 2008), as wells as reduce mPFC GABAa receptor binding (Caldji et al., 2000), further substantiating evidence for a transient dysbalance in small neuronal feedback loops, and potentially providing a substrate for the development of dysfunctional large-scale neuronal networks. Work in the classical rodent models has substantiated these findings of altered PFC development by ELS, reporting on alterations in both dendritic length and regional spine density depending on the age (developmental stage) and the molecular layer in which they are assessed. Mild PS was found to increase spine density in layer III cingulate cortex neurons at weaning (Mychasiuk et al., 2012), and a mild postnatal stressor caused similar effects assessed pre-puberty (PND35; Monroy et al., 2010). However, in adulthood, PS was found to reduce spine density and dendritic branching and length in dACC and orbitofrontal cortex layer II/III pyramidal neurons (Murmu et al., 2006), and to reduce the ratio of mushroom spines; the type forming the most strong and stable synapses (Michelsen et al., 2007). ESD was also found to reduce apical dendritic length in several PFC subregions in the adult offspring, and reduce spine density in frontal cortex layer III neurons (Monroy et al., 2010; Romano-López et al., 2016). Reduced local expression of BDNF mRNA in adult MS (Roceri et al., 2004), LN (Roth et al., 2009), and PS offspring due to increased *Bdnf* DNA methylation [associated with an increase in DNA methyltransferase 1 (DNMT1) expression; Roth et al., 2009; Dong et al., 2015], may relate to these changes. However, findings are not indisputable (Muhammad et al., 2012; Boersma et al., 2014), and it has been suggested that the extent and direction of the effects of ELS on frontal neuronal morphology may depend on the developmental status of the neuronal layer at the time the stress is experienced. ESD on PND1-3 was shown to decrease dendritic spine density in layer II/III neurons of the ACC, but failed to have an effect when applied on PND5-7. ESD on PND14-16 however *increased* spine density on these neurons (Bock et al., 2005). Conversely, ESD on PND5-7 reduced spine density on layer V pyramidal neurons, whereas ESD during the other time intervals did not induce any effects (Gos et al., 2008). As pyramidal cells in layers V/VI are ontogenetically older and therefore establish their synaptic connections earlier than layer II/III pyramidal neurons (Zhang, 2004), these neuron-specific responses to ELS may be due to their different degree of maturity at the time the stressor is experienced. The differential innervation and receptor patterns amongst neuronal layers may be an alternative explanation for their differential sensitivity toward stress (e.g., Zilles et al., 1993). Furthermore, the effects of stress exposure might critically depend on its intensity. Supporting this idea, stressor intensity was found to critically modulate frontal cortex global methylation levels; mild PS increased overall methylation levels at PND21, whereas intense PS induced the opposite effect (Mychasiuk et al., 2011).

Prefrontal CRHR1s have been associated with anxiety (Sotnikov et al., 2014), contribute to the HPA-axis stress response (Jaferi and Bhatnagar, 2007) and were recently found to mediate acute stress-induced executive dysfunction (Uribe-Marino et al., 2016). MS has been observed to significantly increase CRHR1 protein levels in response to acute stress, compared to non-MS controls (O'Malley et al., 2011). However, reported effects of MS on basal CRHR1 expression levels have been mixed, with both no effects (O'Malley et al., 2011) and decreases reported (Ladd et al., 2005). Importantly, ELS reduces GR expression in the PFC, and thereby compromises its negative feedback function on the HPA-axis (Diorio et al., 1993). PS was shown to reduce PFC GR protein levels (Green et al., 2011; Bingham et al., 2013) and binding capacity (McCormick et al., 1995). Also LN and MS induce a significant reduction in PFC GR density (Avishai-Eliner et al., 2001; Ladd et al., 2004, 2005), although this effect is not consistently observed (Huot et al., 2004). Interestingly, a study in which monkeys were prenatally treated with the synthetic glucocorticoid dexamethasone did not observe a decrease in GR expression (Heijtz et al., 2010), suggesting that increased corticosterone exposure in itself is not sufficient to establish these effects. Instead, findings in MS pups of which the dam was given a foster nest for the duration of the separation period implicated a critical role for maternal stress (and potentially care) in influencing GR expression; MS pups from a dam with a foster nest showed increased instead of decreased GR density, accompanied by an (albeit partial) restoration of PVN CRH mRNA levels and ACTH response to stress (Huot et al., 2004). Future research should investigate the exact aspects of the maternal behavior that mediate these normative effects.

## Early life stress effects in a “matching” stressful adult environment

Although findings on basal neuroendocrine function as a consequence of ELS are rather inconsistent, general consensus points toward exaggerated neuroendocrine responses upon the encounter of an acute stressor in most ELS models. Such increased responsivity to environmental challenges is typically considered to be maladaptive, as maladaptation is often defined as deviation from the norm. However, we would like to argue that this increased environmental sensitivity can be both adaptive and maladaptive depending on the context at which it is displayed. Enhanced attention to threat (Pollak and Tolley-Schell, 2003; Shackman et al., 2007) for example, might be very adaptive in dangerous environments, but maladaptive in a save, non-threatening context. Evidence supporting this interpretation of an adaptive role for ELS in case of exposure to adult life stressors has been accumulating. Offspring receiving relatively poor maternal care (low licking/grooming) not only displays impaired spatial learning (Liu et al., 2000b), deficits in long term neutral memory (Bredy et al., 2003), and increased acoustic startle and pre-pulse inhibition (PPI) (Daskalakis et al., 2012), but also *enhanced* memory for stressful events (i.e., contextual fear-conditioning; Champagne et al., 2008; Bagot et al., 2009). Similarly, MD offspring showed impaired spatial learning in the water maze (Oomen et al., 2010), but *improved* cue fear-conditioned memory (Oomen et al., 2011) and contextual learning in a high-stress environment (Oomen et al., 2010). In line with this, mild ELS reduced responsiveness to acute stress exposure (acoustic and restraint) in terms of corticosteroid release and reduction in body weight (Kiank et al., 2009). Some studies even indicated anxiolytic effects of mild-moderate ELS (Cannizzaro et al., 2006; Ehrlich and Rainnie, 2015). Besides this enhanced coping with acutely stressful conditions, ELS also seems to “protect” against the detrimental effects of prolonged stress exposure in adulthood. Isolation rearing was shown to significantly disrupt PPI in control animals but not in those that were maternally deprived (Ellenbroek and Cools, 2002), and to more severely affect anxious, social, and depressive phenotypes in controls compared to LN offspring (Santarelli et al., 2014). Moreover, offspring that received enhanced maternal care (high licking/grooming) reared in isolation displayed lower PPI levels and the highest apomorphine-induced gnawing, a measure marking psychosis susceptibility, compared to offspring that received low maternal care (Daskalakis et al., 2012). Even the effects of severe prolonged stress (i.e., 24 days of chronic unpredictable stress) were buffered by ELS; whereas this stressor induced a significant impairment in contextual fear memory in control animals, stressed MD rats displayed similar performance to non-stressed control animals (Zalosnik et al., 2014). All these findings seem to support the match-mismatch theory, proposing adaptive effects of ELS exposure in a matching stressful environment in adulthood. Importantly, the adaptive potential of ELS seems to interact with an individual's programming sensitivity (or early plasticity), which might be determined by three factors; heritable variation, developmental experience, and the timing of the experience (Nederhof and Schmidt, 2012). Animals exposed to inescapable shock stress for example showed reduced escape latencies to escapable stress when they experienced MS early in life, and this effect was more pronounced in animals with reduced expression levels of the serotonin transporter (van der Doelen et al., 2013), supposedly reflecting higher susceptibility to environmental factors (“programming sensitivity”). Another study showed that MS decreased anxiety- and depressive-like behaviors and enhanced social interaction in rats with heightened inborn stress-susceptibility (i.e., Wistar–Kyoto rats), whereas MS induced opposite effects in Wistar MS offspring (Rana et al., 2015).

Similar protective effects of ELS to stress exposure in adulthood are emerging in terms of neuroendocrine responding. While MS seems to result in significantly higher corticosterone stress responses in adulthood (Ladd et al., 2004, 2005; Plotsky et al., 2005; Lippmann et al., 2007; Lajud et al., 2012), the additional experience of chronic stress in adult life has been shown to normalize these responses to the level observed in non-stressed non-MS controls (Ladd et al., 2005) or to even reduce basal ACTH and corticosterone levels compared to stressed non-MS animals (Renard et al., 2007). These effects were associated with an upregulation in hippocampal GR expression, and a normalization of GR levels in the PVN (Renard et al., 2010). Moreover, exposure to chronic stress reduced amygdala CRH mRNA expression in MS offspring and did not induce an increase in PVN expression levels (as observed in the stressed control animals; Ladd et al., 2005). At the same time, acute stress exposure reduced PVN CRHR1 mRNA expression specifically in MS animals, returning them to similar levels as those in non-MS controls (O'Malley et al., 2011). In terms of the HPA-axis' external modulators, PS has been found to protect rats from the degenerating effects of chronic stress on spine density and morphology (reducing the density of mushroom spines in particular) of mPFC neurons (Michelsen et al., 2007). Additionally, MS was shown to prevent the observed reduction in mPFC BDNF mRNA expression in response to acute stress (Roceri et al., 2004). Furthermore, exposure of MS animals to chronic stress later in life was shown to significantly increase frontal cortex GR mRNA expression levels, eliminating the significant difference in expression with the non-stressed non-MS controls (Ladd et al., 2005). In the hippocampus, corticosterone administration enhanced CA1 LTP in offspring that received low maternal care, whereas significant impairments due to corticosterone were observed in high licking/grooming (LG) offspring (Champagne et al., 2008). This CORT-induced impairment in LTP in the high LG offspring was associated with increased NMDA receptor function in these animals, and was also observed in low LG offspring under basal conditions (Bagot et al., 2012). Moreover, corticosterone was shown to enhance DG LTP in MS animals compared to their non-MS controls, even though the MS caused a reduction of neurogenesis and an altered dendritic complexity (Oomen et al., 2010). These studies imply that chronic stress in adulthood actually restores PFC and hippocampal function and their inhibition of the HPA-axis.

Although still preliminary, first evidence in humans supporting the adaptive effects of early life adversity under matching situations later in life has also become apparent. Stress during pregnancy was shown to be a consistent predictor of cortisol reactivity in infants; although PS increased overall basal cortisol levels in children, it decreased cortisol reactivity to maternal separation (Tollenaar et al., 2011). Another study showed that moderate ELS was associated with lower implicit anxiety than low ELS (Edge et al., 2009), whereas neuroimaging work indicated that ELS was not only associated with a reduced cortisol response to psychosocial stress, but also with an attenuated stress-induced limbic deactivation, reflecting relative stress resilience (Grimm et al., 2014).

However, the interaction between stress in early life and adulthood is not always as straightforward. Acute stress exposure was also shown to induce an increase in prefrontal CRHR1 expression in MS animals (compared to a decrease in controls), but not a reduction in amygdala CRHR1 expression (as seen in controls), and to induce significant increased amygdalar CRHR2 and hippocampal CRHR1 expression in MS animals specifically (O'Malley et al., 2011). Moreover, chronic stress has been shown to add to the effects of MS in terms of decreasing CRHR1 mRNA expression in the frontal and parietal cortex, whereas it normalized CRHR1 binding potential in these regions to the level of non-stressed controls (Ladd et al., 2005). The exact meaning of these findings should be assessed in further studies. Moreover, in contrast to these adaptive/protective effects of ELS, other findings support the so-called “two/three-hit hypothesis,” in which later life stressors worsen the effects of ELS and genetic predispositions sensitize an animal to these detrimental effects (Daskalakis et al., 2013). For example, a combination of MS and later life corticosterone treatment or chronic stress exaggerated the impairing effects of either treatment alone on learning and memory, PPI, and hippocampal BDNF expression (Choy et al., 2008, 2009; Llorente et al., 2011). Moreover, PS appeared to increase vulnerability to chronic restraint stress in adulthood, elevating anxiety and basal hypothalamic CRH and ACTH levels, although basal corticosteroid levels were remarkably reduced (Chung et al., 2005). Behaviorally, MS animals were found more sensitive to social-defeat anhedonia (Der-Avakian and Markou, 2010), and to display elevated corticosterone stress responses and increased depression-like behavior as a consequence of repeated restraint stress in adulthood (Uchida et al., 2010). In line with these behavioral findings, MS was shown to decrease overall hippocampal GR levels (Aisa et al., 2007, 2008), and even more so in case of adult chronic stress (Aisa et al., 2008).

Finally, more ambiguous findings in support of both theories have been reported. Chronic stress exposure induced a smaller reduction in CA3 dendritic length and a blunted response on thymus and adrenal weight in MS animals compared to controls (Eiland and McEwen, 2012). However, at the same time, MS animals displayed increased chronic stress-induced anxiety and novelty-induced corticosterone secretion (Eiland and McEwen, 2012). Again, it seems important to note that all of the abovementioned phenotypic alterations should be interpreted in the light of the specific environmental context. While deviations from the norm (such as increased anxiety and heightened corticosterone responses) are usually seen as maladaptive, they could be highly adaptive if the context requires. Moreover, besides the test context, the exact extent of programming by ELS may critically determine how individuals function in adult life. The match-mismatch theory may apply to individuals that are sensitively programmed (by a combination of genetic makeup and strong developmental experience at a vulnerable time point in development) for an adverse environment, while the cumulative stress (2/3-hit) hypothesis might apply to individuals that did not undergo such strong programming effects (Nederhof and Schmidt, 2012).

## Conclusion and discussion

As described in this review, ELS induces enduring neuroplasticity of the HPA-axis by influencing the developmental trajectories of brain maturation, and exerting a wide range of long-lasting effects, encompassing alterations in neuroendocrine signaling, neuronal morphology and plasticity, and regional brain volume and function. Both PS and MS seem to induce a hyper-responsive HPA-axis, boosting the amygdala's excitatory drive, while impairing regulatory negative feedback function of the hippocampus and PFC. Human findings are in line with such HPA-axis hyperactivity due to prenatal and “mild-moderate” neonatal stress. Prenatal stress and anxiety were shown to increase stress-induced cortisol responses in pre-adolescent children (Gutteling et al., 2005; O'Connor et al., 2005), and also adversity in early life (e.g., interparental aggression, corporal punishment, or frequent emotional maternal withdrawal) was found to increase basal cortisol levels (Davies et al., 2009), as well as cortisol stress responses (Bugental et al., 2003). Conversily, child-reported maternal warmth predicted lower cortisol stress responses (Luecken et al., 2016). However, more severe stress, ESD, seems to exert different effects as stress-induced corticosterone levels in ESD animals are either unaffected or decreased as compared to non-stressed controls. Similarly, studies in human children exposed to severe stress (due to severe neglect, abuse, or deprivation e.g., in orphanages or institutions, or involvement with child protective services) report on lower basal levels of corticosteroids (e.g., Carlson and Earls, 1997; Gunnar and Donzella, 2002; Bruce et al., 2009; Bernard et al., 2015). This hypocortisolism might either be caused by a reduced response of the pituitary to the CRH-drive from the hypothalamus (Fries et al., 2005) or by target tissue hypersensitivity to corticosteroids (Yehuda et al., 2006). Interestingly, hypocortisolism is also observed in PTSD patients, in combination with increased glucocorticoid sensitivity (Rohleder et al., 2004). However, similar to the described preclinical rodent studies, many discrepancies regarding altered HPA-axis function as observed in ELS-related psychopathology remain. Future dedicated research into the exact nature, duration, and developmental period affected by the early life adversity may shed light on these obscurities.

Overall, many conflicting results have been reported for the effects of ELS in rodents. Results may vary due to the use of different stressors, their distinct severity, and differential duration and frequency. Moreover, differences in testing conditions, such as the time of the day (influencing concurrent circulating corticosterone levels; Dickmeis, 2009), or relatively stressful context of testing or sacrifice may affect the outcome. Furthermore, the effects of stress exposure may critically depend on the (additive or compensatory changes in) alterations in maternal care caused by the stressor (Box 2). As the developmental trajectories of brain regions and systems are affected (either delayed or characterized by temporary attempts to compensation), the age at which ELS effects are assessed is also a critical factor. Moreover, gene x environment interactions (Nugent et al., 2011) may underlie the differential effects observed for different strains of the animals (e.g., Long Evans, Wistar, Sprague-Dawley, Brattleboro rats, CD1, C57BL/6J, C57Bl/6N, BALB/C mice). Another important factor is sex (see Box 3). Sexually dimprophic gonadal hormones critically interact with the stress response (reviewed in Kajantie and Phillips, 2006). The biological substrates of sex dimorphisms pertaining to stress however remain understudied and require further investigation. Lastly, the exact outcome of stress exposure seems to depend on the maturational status of a given brain region at the time of the stressor, e.g., the experience of adversity at times of frontal cortex development induce differential effects from those experienced during those of the hippocampus or amygdala (Lupien et al., 2009). In line with this, experiments in rats revealed that MS between PND2-20 was shown to exert negative effects on the spine density in hippocampus (Andersen and Teicher, 2004), whereas stress experienced later in development, i.e., PND30-35, affected synaptic density in the prefrontal cortex (Leussis et al., 2008). Findings in humans further corroborate this by showing that the repeated experience of sexual abuse was related to decreased hippocampal volume when it occurred early in childhood, but with reduced prefrontal cortex volume if it occurred during adolescence (Teicher et al., 2006; Andersen et al., 2008). Similarly, the psychopathology developed as a consequence of ELS may depend on the developmental stage affected. Women were for example shown to display increased risk for major depression when they experienced a trauma before the age of 12, but to PTSD when the trauma occurred between 12 and 18 years of age (Maercker et al., 2004). As the hippocampus in humans develops till 2 years of age, whereas that of the amygdala continues until the late 20 s and that of the frontal cortex primarily takes place between 8 and 14 years of age (Giedd et al., 1996), the hippocampus might be the brain area most vulnerable to the effects of stress early in life.

### Concluding

Thus, stress exposure during early life can have severe consequences on our health during later life and increase susceptibility to psychopathology. However, the severe, long-lasting changes in the reactivity of the HPA-axis to stress are not necessarily maladaptive. In this review we point toward several factors that seem to be highly relevant in determining the eventual outcome. Firstly, the nature and timing and duration (Andersen, 2003) of the stressor in combination with the genetic background of the individual, determine how well an individual can adapt to it. Secondly, it depends on the specific endophenotype tested and the context in which it is assessed. High levels of anxiety could for example be adaptive in certain environmental context, whereas impaired spatial memory is not. The latter suggests that even within the same individual evidence for the match/mismatch and cumulative stress hypothesis can be obtained.

## Author contributions

MvB and MH have reviewed literature and wrote the manuscript. JH read and revised the manuscript.

## Funding

This work was supported by the Netherlands Organization for Scientific Research (NWO), grant #864.10.003 awarded to JH and Veni grant #863.15.008 awarded to MH.

### Conflict of interest statement

The authors declare that the research was conducted in the absence of any commercial or financial relationships that could be construed as a potential conflict of interest.
