# Supplementary material for: Modulation of the Hypothalamic-Pituitary-Adrenal Axis by Early Life Stress Exposure
Source: Front Cell Neurosci. 2017 Apr 19;11:87. doi: 10.3389/fncel.2017.00087 (PMC5395581; doi:10.3389/fncel.2017.00087)
Supplement: Supplementary file 1 [file Table1.pdf]

Supplementary Material

Modulation of the Hypothalamic-Pituitary-Adrenal Axis by Early Life Stress Exposure

Miranda van Bodegom, Judith R. Homberg, and Marloes J. A. G. Henckens *

Department of Cognitive Neuroscience, Centre for Neuroscience, Donders Institute for Brain, Cognition and Behaviour, Radboudumc, Nijmegen, Netherlands

*** Correspondence:**
Marloes J.A.G. Henckens: marloes.henckens@radboudumc.nl

SUPPLEMENTARY TABLE 1 | Overview of the effects of early life stress exposure as observed in adulthood in key regions of the HPA-axis

		Prenatal stress	References	Neonatal stress	References
**Hypothalamus**	*Morphology*	Cell proliferation ↓	(Baquedano et al., 2011)	Cell surviving enzymes & proteins ↑	(Irles et al., 2014)
		Apoptosis ↓	(Baquedano et al., 2011)		
				Apoptosis stimulating enzymes & proteins ↓	(Irles et al., 2014)
				Neuronal density ↑	(Irles et al., 2014)
	Neuro-endocrine	Basal CRH – / ↑	(Table 1)	Basal CRH ↑^{MS}/ –$^{\text{MS \& LN \& ESD}}$ /↓^{LN}	(Table 1)
		Stress CRH –♂ / ↑♂♀	(Table 1)	Stress CRH ↑^{MS} /–$^{\text{MS \& ESD}}$	(Table 1)
		CRHR1 ↑♂ / –♂♀ / ↓♀	(Fan et al., 2009; Zohar and Weinstock, 2011; Wang et al., 2013b)	Basal CRHR1 ↑♂	(Bravo et al., 2011; O'Malley et al., 2011)
				Stress-induced CRHR1 –♂	(Bravo et al., 2011; O'Malley et al., 2011)
		CRHR2 – / ↓	(Fan et al., 2009; Zohar and Weinstock, 2011; Wang et al., 2013b)	Basal CRHR2 –♂	(Bravo et al., 2011; O'Malley et al., 2011)
				Stress CRHR2 –♂	(Bravo et al., 2011; O'Malley et al., 2011)
		GR density – / ↓	(McCormick et al., 1995; Bingham et al., 2013)	GR mRNA expression – / ↓	(Workel et al., 2001; Arnett et al., 2015)
				GR binding ↓	(Workel et al., 2001)
**Anterior pituitary**	*Morphology*	Cell proliferation ↓	(Baquedano et al., 2011)		
		Apoptosis ↓	(Baquedano et al., 2011)		
	Neuro-endocrine	Basal ACTH –/↑♀	(Table 2)	Basal ACTH –$^{\text{MS \& ESD}}$	(Table 2)
		Stress ACTH ↓/–/↑♂ / ↑♀	(Table 2)	Stress ACTH –$^{\text{MS \& ESD}}$ /↑^{MS}	(Table 2)
		Basal POMC –	(Brunton and Russell, 2010)	Basal POMC ↑	(Murgatroyd et al., 2009)
		Stress POMC –♀/↑♂	(Brunton and Russell, 2010)	CRHR binding capacity ↓$^{\text{ESD \& LN}}$	(Ladd et al., 1996; Avishai-Eliner et al., 2001)
		CRHR1 ↑	(Fan et al., 2009)		
		CRHR2 ↑	(Fan et al., 2009)		
**Amygdala**	*Morphology*	Volume* ↑/–	(Salm et al., 2004; Kraszpulski et al., 2006)	Branch length –	(Krugers et al., 2012)
				Branch points number –	(Krugers et al., 2012)
		Neuronal cell number* –/↑	(Salm et al., 2004; Kraszpulski et al., 2006)	Dendritic complexity –	(Krugers et al., 2012)
		Glial cell number* –/↑	(Salm et al., 2004; Kraszpulski et al., 2006)	Myelination accelerated (in 5-week old mice)	(Ono et al., 2008)
				Parvalbumin neurons (in peri-adolescent mice) ↑	(Giachino et al., 2007; Seidel et al., 2008)
	Epigenetics			*Crh* gene promoter methylation following repeated adult stress ↑	(van der Doelen et al., 2015)

(Continued)

SUPPLEMENTARY TABLE 1 | Continued

		Prenatal stress	References	Neonatal stress	References
**Amygdala**	*Neuro-endocrine*	Basal *Crh* mRNA ↑	(Brunton and Russell, 2010)	Basal *Crh* mRNA – ♂	(Bravo et al., 2011)
		Local CRH release ↑	(Cratty et al., 1995)	Stress *Crh* mRNA ↑ ESD	(Barna et al., 2003)
		CRHR1 ↑♂*♀*	(Brunton et al., 2011)	*Crhr1* mRNA ↑*♂	(Bravo et al., 2011)
		CRHR2 ↓♂*/↑♀*	(Brunton et al., 2011)	Basal CRHR1 – ♂	(O'Malley et al., 2011)
		GR binding ↑	(McCormick et al., 1995)	Stress CRHR1 ↓ ♂	(O'Malley et al., 2011)
		GR mRNA ↑	(Brunton and Russell, 2010)	*Crhr2* mRNA ↓*♂	(Bravo et al., 2011)
				Basal CRHR2 – ♂	(O'Malley et al., 2011)
				Stress CRHR2 ↑ ♂	(O'Malley et al., 2011)
				Basal GR ↓#	(Arnett et al., 2015)
				GABAa receptor binding ↓*	(Caldji et al., 2000)
	Function	Anxiety ↑	(Estanislau and Morato, 2005; Patin et al., 2005; Abe et al., 2007; Bosch et al., 2007; Morley-Fletcher et al., 2011; Sadler et al., 2011; Schulz et al., 2011; de Souza et al., 2013)	Anxiety ↑	(Huot et al., 2002; Kalinichev et al., 2002; Romeo et al., 2003; Daniels et al., 2004; Rees et al., 2006; Aisa et al., 2007; Lee et al., 2007; Trujillo et al., 2016)
		Depression-like behavior	(Yang et al., 2006; Abe et al., 2007; Sowa et al., 2015)	Depression-like behavior	(Aisa et al., 2007; Lee et al., 2007; Aisa et al., 2008; Hui et al., 2011)
		Cerebral activity ↑	(Laviola et al., 2004)		
**Hippocampus**	*Morphology*	Volume ↓	(Mandyam et al., 2008)	Volume – MS / ↓ LN*	(Huot et al., 2002; Fabricius et al., 2008; Hui et al., 2011; Zalosnik et al., 2014; Naninck et al., 2015)
		Neurogenesis ↓♂/– ♀	(Lemaire et al., 2000; Mandyam et al., 2008; Zuena et al., 2008; Morley-Fletcher et al., 2011; Belnoue et al., 2013; Madhyastha et al., 2013)	Neurogenesis↓♂ MS & LN/–♀ MS & LN	(Oomen et al., 2010; Oomen et al., 2011; Lajud et al., 2012; Naninck et al., 2015)
		Cell proliferation ↓	(Lemaire et al., 2000; Mandyam et al., 2008; Morley-Fletcher et al., 2011; Belnoue et al., 2013)	Cell proliferation ↓ MS*	(Mirescu et al., 2004; Oomen et al., 2010; Hulshof et al., 2011)
		Cell death –♂♀/↑♂	(Lemaire et al., 2000; Mandyam et al., 2008)	DG cell number ↓ MS & ESD	(Leventopoulos et al., 2007; Fabricius et al., 2008; Oomen et al., 2011)
		CA1 & CA3 spine density ↓	(Ishiwata et al., 2005; Martínez-Téllez et al., 2009)	CA1 spine density ↓	(Monroy et al., 2010)

(Continued)

SUPPLEMENTARY TABLE 1 | Continued

		Prenatal stress	References	Neonatal stress	References
**Hippocampus**	*Morphology*	CA3 synapse density ↓	(Ishiwata et al., 2005)	Mossy fiber density ↓ *****	(Huot et al., 2002)
		Dendritic length ↓*	(Hosseini-sharifabad and Hadinedoushan, 2007; Martínez-Téllez et al., 2009)	Dendritic length –$^{\text{MS \& LN}}$/↓$^{\text{LN \& ESD}}$	(Ivy et al., 2010; Monroy et al., 2010; Oomen et al., 2011)
		Dendritic branching ↓*	(Hosseini-sharifabad and Hadinedoushan, 2007)		
		NR1 subunit of NMDAR ↓	(Son et al., 2006)		
		NR2B subunit of NMDAR ↓	(Son et al., 2006)		
	Epigenetics	*Bdnf* exon IV methylation ↓	(St-Cyr and McGowan, 2015)	*Crh* gene promoter methylation ↓*	(Wang et al., 2014)
		Overall *Bdnf* methylation ↓	(Dong et al., 2015)	*Crh* promoter histone 3 acetylation ↑	(Wang et al., 2014)
		DNA methyltransferase 1 expression ↑	(Dong et al., 2015)		
	Neuro-endocrine	GR density ↓	(Henry et al., 1994; Barbazanges et al., 1996; Koehl et al., 1999; Szuran et al., 2000; Chung et al., 2005; Van Waes et al., 2006; Mueller and Bale, 2008; Green et al., 2011; Bingham et al., 2013)	GR mRNA ↓^{MS} / – $^{\text{MS\&LN}}$	(Ladd et al., 2004; Brunson et al., 2005; Ladd et al., 2005; Arnett et al., 2015)
				GR ↓	(Weaver et al., 2004; Aisa et al., 2007; Aisa et al., 2008; Batalha et al., 2013)
		MR<br>• mRNA ↓<br>• density ↓<br>• binding capacity ↓	(Henry et al., 1994; Maccari et al., 1995; Koehl et al., 1999; Van Waes et al., 2006; Brunton and Russell, 2010)	MR mRNA –/↑	(Workel et al., 2001; Ladd et al., 2004; Ladd et al., 2005; Batalha et al., 2013)
		Bdnf mRNA ↓ ***/ –***$^{♂}$	(St-Cyr and McGowan, 2015; Dong et al., 2015)	*Bdnf* mRNA –/↓/↑	(Kuma et al., 2004; Roceri et al., 2004; Greisen et al., 2005; Aisa et al., 2009)
		BDNF ↑$^{♂}$***/–***$^{♀}$	(Zuena et al., 2008)	*Crh* mRNA –/↑	(Bravo et al., 2011; Wang et al., 2014)
				CRH ↑	(Wang et al., 2014)
				CRH expressing interneurons ↑*^{LN}	(Ivy et al., 2010)
				Crhr1 mRNA – $^{♂}$	(Bravo et al., 2011)
				Basal CRHR1 – $^{♂}$	(O'Malley et al., 2011)
				Stress CRHR1 ↑ $^{♂}$	(O'Malley et al., 2011)

(Continued)

SUPPLEMENTARY TABLE 1 | Continued

		Prenatal stress	References	Neonatal stress	References
**Hippocampus**	*Neuro-endocrine*			*Crhr2* mRNA ↓$^{♂}$	(Bravo et al., 2011)
				Basal CRHR2 – $^{♂}$	(O'Malley et al., 2011)
				Stress CRHR2 – $^{♂}$	(O'Malley et al., 2011)
	Function	LTP* ↓	(Yang et al., 2007)	LTP* ↓$^{\text{LN \& MS}}$ / – $^{\text{MS♀ \& ESD}}$ / ↑^{ESD}	(Kehoe and Bronzino, 1999; Brunson et al., 2005; Cui et al., 2006; Ivy et al., 2010; Oomen et al., 2011; Batalha et al., 2013; Wang et al., 2013a; Xiong et al., 2014)
		LTD* ↑	(Yang et al., 2007)		
		Spatial learning ↓	(Lemaire et al., 2000; Ishiwata et al., 2005; Yang et al., 2007; Lui et al., 2011; Schulz et al., 2011)	Spatial learning ↓ $^{\text{MS \& ESD}}$	(Huot et al., 2002; Aisa et al., 2007; Garner et al., 2007; Kosten et al., 2007; Zhu et al., 2010; Hulshof et al., 2011; Tata et al., 2015; Wang et al., 2015)
		Spatial memory ↓	(Lemaire et al., 2000; Yang et al., 2007; Lui et al., 2011; Schulz et al., 2011)	Spatial memory ↓$^{\text{MS \& LN}}$	(Brunson et al., 2005; Rice et al., 2008; Ivy et al., 2010; Zhu et al., 2010; Hui et al., 2011; Tata et al., 2015)
**Prefrontal cortex**	*Morphology*	Spine density ↓	(Murmu et al., 2006; Muhammad et al., 2012)	Spine density ↑ ^{MS}* / ↓ ^{ESD}	(Monroy et al., 2010; Muhammad et al., 2012)
		Dendritic branching ↑/↓	(Murmu et al., 2006; Muhammad et al., 2012)	Dendritic branching **–** ^{ESD}/ ↑ ^{MS}	(Monroy et al., 2010; Muhammad et al., 2012)
		Dendritic length ↑/↓	(Murmu et al., 2006; Muhammad et al., 2012)	Dendritic length ↑ *^{MS} / ↓ ^{ESD}	(Monroy et al., 2010; Muhammad et al., 2012; Romano-Lopez et al., 2016)
		Mushroom spine ratio ↓	(Michelsen et al., 2007)	*Bdnf* mRNA ↓$^{\text{MS \& LN}}$	(Roceri et al., 2004; Roth et al., 2009)
		Bdnf mRNA –/↓	(Boersma et al., 2014; Dong et al., 2015)		
	Epigenetics	Overall *Bdnf* methylation ↑	(Dong et al., 2015)	*Bdnf* methylation ↑ ^{LN}	(Roth et al., 2009)
		Bdnf exon IV methylation –	(Boersma et al., 2014)		
		Bdnf exon VI methylation ↓	(Boersma et al., 2014)		
		DNA methyltransferase 1 expression ↑	(Dong et al., 2015)		

(Continued)

SUPPLEMENTARY TABLE 1 | Continued

		Prenatal stress	References	Neonatal stress	References
**Prefrontal cortex**	*Neuro-endocrine*	GR binding ↓	(McCormick et al., 1995)	Basal *Crhr1* mRNA ↓	(Ladd et al., 2005)
		GR protein ↓	(Green et al., 2011; Bingham et al., 2013)	Basal CRHR1 ↓ / –	(Ladd et al., 2005; O'Malley et al., 2011)
				Stress CRHR1 ↑*	(O'Malley et al., 2011)
				GR density ↓ $^{MS \& LN}$ / – ^{MS}	(Avishai-Eliner et al., 2001; Huot et al., 2004; Ladd et al., 2004; Ladd et al., 2005)
	Function	LTP ↓	(Sowa et al., 2015)	LTP IL layer II/III-layer V ↓	(Xiong et al., 2014)
		EPSCs layer II/III pyramidal neurons ↑	(Sowa et al., 2015)	Regional CBF ↓*	(Sadler et al., 2011)

↓ *indicates a significant decrease,* ↑ *a significant increase, and - no significant difference. In case no specific neonatal stress model is mentioned, data apply to maternal separation. If no sex is specified, no sex-specific effects are reported.* ♂ *results in males;* ♀ *results in females; * subregion specific;* # *species-specific; ACTH, adrenocorticotropic hormone; BDNF, brain-derived neurotrophic factor; CRH, corticotrophin-releasing factor; CRHR1, CRH receptor 1; CRHR2, CRH receptor 2; DG, dentate gyrus; ESD, early social deprivation; GR, glucocorticoid receptor; LTD, long-term depression; LTP, long-term potentiation; LN, limited nesting; MR, mineralocorticoid receptor; MS, maternal separation; POMC, Proopiomelanocortin.*

References

Abe, H., Hidaka, N., Kawagoe, C., Odagiri, K., Watanabe, Y., Ikeda, T., et al. (2007). Prenatal psychological stress causes higher emotionality, depression-like behavior, and elevated activity in the hypothalamo-pituitary-adrenal axis. *Neuroscience Research* 59(2)**,** 145-151. doi: http://dx.doi.org/10.1016/j.neures.2007.06.1465.

Aisa, B., Elizalde, N., Tordera, R., Lasheras, B., Del Río, J., and Ramírez, M.J. (2009). Effects of neonatal stress on markers of synaptic plasticity in the hippocampus: implications for spatial memory. *Hippocampus* 19(12)**,** 1222-1231. doi: 10.1002/hipo.20586.

Aisa, B., Tordera, R., Lasheras, B., Del Río, J., and Ramírez, M.J. (2007). Cognitive impairment associated to HPA axis hyperactivity after maternal separation in rats. *Psychoneuroendocrinology* 32(3)**,** 256-266. doi: 10.1016/j.psyneuen.2006.12.013.

Aisa, B., Tordera, R., Lasheras, B., Del Río, J., and Ramírez, M.J. (2008). Effects of maternal separation on hypothalamic–pituitary–adrenal responses, cognition and vulnerability to stress in adult female rats. *Neuroscience* 154(4)**,** 1218-1226. doi: http://dx.doi.org/10.1016/j.neuroscience.2008.05.011.

Arnett, M.G., Pan, M.S., Doak, W., Cyr, P.E., Muglia, L.M., and Muglia, L.J. (2015). The role of glucocorticoid receptor-dependent activity in the amygdala central nucleus and reversibility of early-life stress programmed behavior. *Translational Psychiatry* 5**,** e542. doi: 10.1038/tp.2015.35.

Avishai-Eliner, S., Gilles, E.E., Eghbal-Ahmadi, M., Bar-El, Y., and Baram, T.Z. (2001). Altered regulation of gene and protein expression of hypothalamic-pituitary-adrenal axis components in an immature rat model of chronic stress. *J Neuroendocrinol* 13(9)**,** 799-807.

Baquedano, E., Garcia-Caceres, C., Diz-Chaves, Y., Lagunas, N., Calmarza-Font, I., Azcoitia, I., et al. (2011). Prenatal stress induces long-term effects in cell turnover in the hippocampus-hypothalamus-pituitary axis in adult male rats. *PLoS One* 6(11)**,** e27549. doi: 10.1371/journal.pone.0027549.

Barbazanges, A., Piazza, P.V., Le Moal, M., and Maccari, S. (1996). Maternal glucocorticoid secretion mediates long-term effects of prenatal stress. *J Neurosci* 16(12)**,** 3943-3949.

Barna, I., Bálint, E., Baranyi, J., Bakos, N., Makara, G.B., and Haller, J. (2003). Gender-specific effect of maternal deprivation on anxiety and corticotropin-releasing hormone mRNA expression in rats. *Brain Research Bulletin* 62(2)**,** 85-91. doi: http://dx.doi.org/10.1016/S0361-9230(03)00216-8.

Batalha, V.L., Pego, J.M., Fontinha, B.M., Costenla, A.R., Valadas, J.S., Baqi, Y., et al. (2013). Adenosine A(2A) receptor blockade reverts hippocampal stress-induced deficits and restores corticosterone circadian oscillation. *Mol Psychiatry* 18(3)**,** 320-331. doi: 10.1038/mp.2012.8.

Belnoue, L., Grosjean, N., Ladevèze, E., Abrous, D.N., and Koehl, M. (2013). Prenatal Stress Inhibits Hippocampal Neurogenesis but Spares Olfactory Bulb Neurogenesis. *PLoS ONE* 8(8)**,** e72972. doi: 10.1371/journal.pone.0072972.

Bingham, B.C., Sheela Rani, C.S., Frazer, A., Strong, R., and Morilak, D.A. (2013). Exogenous prenatal corticosterone exposure mimics the effects of prenatal stress on adult brain stress response systems and fear extinction behavior. *Psychoneuroendocrinology* 38(11)**,** 2746-2757. doi: 10.1016/j.psyneuen.2013.07.003.

Boersma, G.J., Lee, R.S., Cordner, Z.A., Ewald, E.R., Purcell, R.H., Moghadam, A.A., et al. (2014). Prenatal stress decreases Bdnf expression and increases methylation of Bdnf exon IV in rats. *Epigenetics* 9(3)**,** 437-447. doi: 10.4161/epi.27558.

Bosch, O.J., Müsch, W., Bredewold, R., Slattery, D.A., and Neumann, I.D. (2007). Prenatal stress increases HPA axis activity and impairs maternal care in lactating female offspring: Implications for postpartum mood disorder. *Psychoneuroendocrinology* 32(3)**,** 267-278. doi: http://dx.doi.org/10.1016/j.psyneuen.2006.12.012.

Bravo, J.A., Dinan, T.G., and Cryan, J.F. (2011). Alterations in the central CRF system of two different rat models of comorbid depression and functional gastrointestinal disorders. *International Journal of Neuropsychopharmacology* 14(5)**,** 666-683. doi: 10.1017/s1461145710000994.

Brunson, K.L., Kramár, E., Lin, B., Chen, Y., Colgin, L.L., Yanagihara, T.K., et al. (2005). Mechanisms of late-onset cognitive decline after early-life stress. *The Journal of neuroscience* 25(41)**,** 9328-9338. doi: 10.1523/JNEUROSCI.2281-05.2005.

Brunton, P.J., Donadio, M.V., and Russell, J.A. (2011). Sex differences in prenatally programmed anxiety behaviour in rats: differential corticotropin-releasing hormone receptor mRNA expression in the amygdaloid complex. *Stress* 14(6)**,** 634-643. doi: 10.3109/10253890.2011.604750.

Brunton, P.J., and Russell, J.A. (2010). Prenatal Social Stress in the Rat Programmes Neuroendocrine and Behavioural Responses to Stress in the Adult Offspring: Sex-Specific Effects. *Journal of Neuroendocrinology* 22(4)**,** 258-271. doi: 10.1111/j.1365-2826.2010.01969.x.

Caldji, C., Francis, D., Sharma, S., Plotsky, P.M., and Meaney, M.J. (2000). The effects of early rearing environment on the development of GABAA and central benzodiazepine receptor levels and novelty-induced fearfulness in the rat. *Neuropsychopharmacology* 22(3)**,** 219-229. doi: 10.1016/S0893-133X(99)00110-4.

Chung, S., Son, G.H., Park, S.H., Park, E., Lee, K.H., Geum, D., et al. (2005). Differential adaptive responses to chronic stress of maternally stressed male mice offspring. *Endocrinology* 146(7)**,** 3202-3210. doi: 10.1210/en.2004-1458.

Cratty, M.S., Ward, H.E., Johnson, E.A., Azzaro, A.J., and Birkle, D.L. (1995). Prenatal stress increases corticotropin-releasing factor (CRF) content and release in rat amygdala minces. *Brain Research* 675(1–2)**,** 297-302. doi: http://dx.doi.org/10.1016/0006-8993(95)00087-7.

Cui, M., Yang, Y., Yang, J., Zhang, J., Han, H., Ma, W., et al. (2006). Enriched environment experience overcomes the memory deficits and depressive-like behavior induced by early life stress. *Neurosci Lett* 404(1-2)**,** 208-212. doi: 10.1016/j.neulet.2006.05.048.

Daniels, W.M.U., Pietersen, C.Y., Carstens, M.E., and Stein, D.J. (2004). Maternal Separation in Rats Leads to Anxiety-Like Behavior and a Blunted ACTH Response and Altered Neurotransmitter Levels in Response to a Subsequent Stressor. *Metabolic Brain Disease* 19(1)**,** 3-14. doi: 10.1023/B:MEBR.0000027412.19664.b3.

de Souza, M.A., Centenaro, L.A., Menegotto, P.R., Henriques, T.P., Bonini, J., Achaval, M., et al. (2013). Prenatal Stress Produces Social Behavior Deficits and Alters the Number of Oxytocin and Vasopressin Neurons in Adult Rats. *Neurochemical Research* 38(7)**,** 1479-1489. doi: 10.1007/s11064-013-1049-5.

Dong, E., Dzitoyeva, S.G., Matrisciano, F., Tueting, P., Grayson, D.R., and Guidotti, A. (2015). Brain-Derived Neurotrophic Factor Epigenetic Modifications Associated with Schizophrenia-like Phenotype Induced by Prenatal Stress in Mice. *Biological Psychiatry* 77(6)**,** 589-596. doi: 10.1016/j.biopsych.2014.08.012.

Estanislau, C., and Morato, S. (2005). Prenatal stress produces more behavioral alterations than maternal separation in the elevated plus-maze and in the elevated T-maze. *Behavioural Brain Research* 163(1)**,** 70-77. doi: http://dx.doi.org/10.1016/j.bbr.2005.04.003.

Fabricius, K., Wörtwein, G., and Pakkenberg, B. (2008). The impact of maternal separation on adult mouse behaviour and on the total neuron number in the mouse hippocampus. *Brain Structure & Function* 212(5)**,** 403-416. doi: 10.1007/s00429-007-0169-6.

Fan, J.M., Chen, X.Q., Jin, H., and Du, J.Z. (2009). Gestational hypoxia alone or combined with restraint sensitizes the hypothalamic–pituitary–adrenal axis and induces anxiety-like behavior in adult male rat offspring. *Neuroscience* 159(4)**,** 1363-1373. doi: http://dx.doi.org/10.1016/j.neuroscience.2009.02.009.

Garner, B., Wood, S.J., Pantelis, C., and van den Buuse, M. (2007). Early maternal deprivation reduces prepulse inhibition and impairs spatial learning ability in adulthood: No further effect of post-pubertal chronic corticosterone treatment. *Behavioural Brain Research* 176(2)**,** 323-332. doi: http://dx.doi.org/10.1016/j.bbr.2006.10.020.

Giachino, C., Canalia, N., Capone, F., Fasolo, A., Alleva, E., Riva, M.A., et al. (2007). Maternal deprivation and early handling affect density of calcium binding protein-containing neurons in selected brain regions and emotional behavior in periadolescent rats. *Neuroscience* 145(2)**,** 568-578. doi: 10.1016/j.neuroscience.2006.12.042.

Green, M.K., Rani, C.S.S., Joshi, A., Soto-Piña, A.E., Martinez, P.A., Frazer, A., et al. (2011). Prenatal stress induces long term stress vulnerability, compromising stress response systems in the brain and impairing extinction of conditioned fear after adult stress. *Neuroscience* 192**,** 438-451. doi: http://dx.doi.org/10.1016/j.neuroscience.2011.06.041.

Greisen, M.H., Altar, C.A., Bolwig, T.G., Whitehead, R., and Wörtwein, G. (2005). Increased adult hippocampal brain-derived neurotrophic factor and normal levels of neurogenesis in maternal separation rats. *Journal of Neuroscience Research* 79(6)**,** 772-778. doi: 10.1002/jnr.20418.

Henry, C., Kabbaj, M., Simon, H., Moal, M., and Maccari, S. (1994). Prenatal stress increases the hypothalamo-pituitary-adrenal axis response in young and adult rats. *Journal of neuroendocrinology* 6(3)**,** 341-345. doi: 10.1111/j.1365-2826.1994.tb00591.x.

Hosseini-sharifabad, M., and Hadinedoushan, H. (2007). Prenatal stress induces learning deficits and is associated with a decrease in granules and ca3 cell dendritic tree size in rat hippocampus. *Anatomical Science International* 82(4)**,** 211. doi: 10.1111/j.1447-073X.2007.00186.x.

Hui, J.-j., Zhang, Z.-j., Liu, S.-s., Xi, G.-j., Zhang, X.-r., Teng, G.-J., et al. (2011). Hippocampal neurochemistry is involved in the behavioural effects of neonatal maternal separation and their reversal by post-weaning environmental enrichment: A magnetic resonance study. *Behavioural Brain Research* 217(1)**,** 122-127. doi: http://dx.doi.org/10.1016/j.bbr.2010.10.014.

Hulshof, H.J., Novati, A., Sgoifo, A., Luiten, P.G.M., den Boer, J.A., and Meerlo, P. (2011). Maternal separation decreases adult hippocampal cell proliferation and impairs cognitive performance but has little effect on stress sensitivity and anxiety in adult Wistar rats. *Behavioural Brain Research* 216(2)**,** 552-560. doi: http://dx.doi.org/10.1016/j.bbr.2010.08.038.

Huot, R.L., Gonzalez, M.E., Ladd, C.O., Thrivikraman, K.V., and Plotsky, P.M. (2004). Foster litters prevent hypothalamic-pituitary-adrenal axis sensitization mediated by neonatal maternal separation. *Psychoneuroendocrinology* 29(2)**,** 279-289. doi: http://dx.doi.org/10.1016/S0306-4530(03)00028-3.

Huot, R.L., Plotsky, P.M., Lenox, R.H., and McNamara, R.K. (2002). Neonatal maternal separation reduces hippocampal mossy fiber density in adult Long Evans rats. *Brain Research* 950(1–2)**,** 52-63. doi: http://dx.doi.org/10.1016/S0006-8993(02)02985-2.

Irles, C., Nava-Kopp, A.T., Morán, J., and Zhang, L. (2014). Neonatal maternal separation up-regulates protein signalling for cell survival in rat hypothalamus. *Stress* 17(3)**,** 275-284. doi: http://dx.doi.org/10.3109/10253890.2014.913017.

Ishiwata, H., Shiga, T., and Okado, N. (2005). Selective serotonin reuptake inhibitor treatment of early postnatal mice reverses their prenatal stress-induced brain dysfunction. *Neuroscience* 133(4)**,** 893-901. doi: 10.1016/j.neuroscience.2005.03.048.

Ivy, A.S., Rex, C.S., Chen, Y., Dubé, C., Maras, P.M., Grigoriadis, D.E., et al. (2010). Hippocampal dysfunction and cognitive impairments provoked by chronic early-life stress involve excessive activation of CRH receptors. *The Journal of Neuroscience* 30(39)**,** 13005-13015. doi: http://dx.doi.org/10.1523/JNEUROSCI.1784-10.2010.

Kalinichev, M., Easterling, K.W., Plotsky, P.M., and Holtzman, S.G. (2002). Long-lasting changes in stress-induced corticosterone response and anxiety-like behaviors as a consequence of neonatal maternal separation in Long–Evans rats. *Pharmacology Biochemistry and Behavior* 73(1)**,** 131-140. doi: http://dx.doi.org/10.1016/S0091-3057(02)00781-5.

Kehoe, P., and Bronzino, J.D. (1999). Neonatal stress alters LTP in freely moving male and female adult rats. *Hippocampus* 9(6)**,** 651-658. doi: 10.1002/(SICI)1098-1063(1999)9:6<651::AID-HIPO6>3.0.CO;2-P.

Koehl, M., Darnaudéry, M., Dulluc, J., Van Reeth, O., Moal, M.L., and Maccari, S. (1999). Prenatal stress alters circadian activity of hypothalamo–pituitary–adrenal axis and hippocampal corticosteroid receptors in adult rats of both gender. *Journal of neurobiology* 40(3)**,** 302-315. doi: 10.1002/(SICI)1097-4695(19990905)40:3<302::AID-NEU3>3.0.CO;2-7.

Kosten, T.A., Karanian, D.A., Yeh, J., Haile, C.N., Kim, J.J., Kehoe, P., et al. (2007). Memory impairments and hippocampal modifications in adult rats with neonatal isolation stress experience. *Neurobiol Learn Mem* 88(2)**,** 167-176. doi: 10.1016/j.nlm.2007.03.011.

Kraszpulski, M., Dickerson, P.A., and Salm, A.K. (2006). Prenatal stress affects the developmental trajectory of the rat amygdala. *Stress* 9(2)**,** 85-95. doi: 10.1080/10253890600798109.

Krugers, H.J., Oomen, C.A., Gumbs, M., Li, M., Velzing, E.H., Joels, M., et al. (2012). Maternal deprivation and dendritic complexity in the basolateral amygdala. *Neuropharmacology* 62(1)**,** 534-537. doi: http://dx.doi.org/10.1016/j.neuropharm.2011.09.022.

Kuma, H., Miki, T., Matsumoto, Y., Gu, H., Li, H.-P., Kusaka, T., et al. (2004). Early maternal deprivation induces alterations in brain-derived neurotrophic factor expression in the developing rat hippocampus. *Neuroscience Letters* 372(1–2)**,** 68-73. doi: http://dx.doi.org/10.1016/j.neulet.2004.09.012.

Ladd, C.O., Huot, R.L., Thrivikraman, K.V., Nemeroff, C.B., and Plotsky, P.M. (2004). Long-term adaptations in glucocorticoid receptor and mineralocorticoid receptor mrna and negative feedback on the hypothalamo-pituitary-adrenal axis following neonatal maternal separation. *Biological Psychiatry* 55(4)**,** 367-375. doi: http://dx.doi.org/10.1016/j.biopsych.2003.10.007.

Ladd, C.O., Owens, M.J., and Nemeroff, C. (1996). Persistent changes in corticotropin-releasing factor neuronal systems induced by maternal deprivation. *Endocrinology* 137(4)**,** 1212-1218. doi: http://dx.doi.org/10.1210/endo.137.4.8625891.

Ladd, C.O., Thrivikraman, K.V., Huot, R.L., and Plotsky, P.M. (2005). Differential neuroendocrine responses to chronic variable stress in adult Long Evans rats exposed to handling-maternal separation as neonates. *Psychoneuroendocrinology* 30(6)**,** 520-533. doi: http://dx.doi.org/10.1016/j.psyneuen.2004.12.004.

Lajud, N., Roque, A., Cajero, M., Gutiérrez-Ospina, G., and Torner, L. (2012). Periodic maternal separation decreases hippocampal neurogenesis without affecting basal corticosterone during the stress hyporesponsive period, but alters HPA axis and coping behavior in adulthood. *Psychoneuroendocrinology* 37(3)**,** 410-420. doi: http://dx.doi.org/10.1016/j.psyneuen.2011.07.011.

Laviola, G., Rea, M., Morley-Fletcher, S., Di Carlo, S., Bacosi, A., De Simone, R., et al. (2004). Beneficial effects of enriched environment on adolescent rats from stressed pregnancies. *European Journal of Neuroscience* 20(6)**,** 1655-1664. doi: 10.1111/j.1460-9568.2004.03597.x.

Lee, J.-H., Kim, H.J., Kim, J.G., Ryu, V., Kim, B.-T., Kang, D.-W., et al. (2007). Depressive behaviors and decreased expression of serotonin reuptake transporter in rats that experienced neonatal maternal separation. *Neuroscience Research* 58(1)**,** 32-39. doi: http://dx.doi.org/10.1016/j.neures.2007.01.008.

Lemaire, V., Koehl, M., Le Moal, M., and Abrous, D.N. (2000). Prenatal stress produces learning deficits associated with an inhibition of neurogenesis in the hippocampus. *Proceedings of the National Academy of Sciences of the United States of America* 97(20)**,** 11032-11037. doi: 10.1073/pnas.97.20.11032.

Leventopoulos, M., Rüedi-Bettschen, D., Knuesel, I., Feldon, J., Pryce, C.R., and Opacka-Juffry, J. (2007). Long-term effects of early life deprivation on brain glia in Fischer rats. *Brain Research* 1142**,** 119-126. doi: http://dx.doi.org/10.1016/j.brainres.2007.01.039.

Lui, C.C., Wang, J.-Y., Tain, Y.-L., Chen, Y.-C., Chang, K.-A., Lai, M.-C., et al. (2011). Prenatal stress in rat causes long-term spatial memory deficit and hippocampus MRI abnormality: Differential effects of postweaning enriched environment. *Neurochemistry International* 58(3)**,** 434-441. doi: http://dx.doi.org/10.1016/j.neuint.2011.01.002.

Maccari, S., Piazza, P.V., Kabbaj, M., Barbazanges, A., Simon, H., and Le Moal, M. (1995). Adoption reverses the long-term impairment in glucocorticoid feedback induced by prenatal stress. *The Journal of Neuroscience* 15(1)**,** 110-116.

Madhyastha, S., Sekhar, S., and Rao, G. (2013). Resveratrol improves postnatal hippocampal neurogenesis and brain derived neurotrophic factor in prenatally stressed rats. *International Journal of Developmental Neuroscience* 31(7)**,** 580-585. doi: http://dx.doi.org/10.1016/j.ijdevneu.2013.06.010.

Mandyam, C.D., Crawford, E.F., Eisch, A.J., Rivier, C.L., and Richardson, H.N. (2008). Stress experienced in utero reduces sexual dichotomies in neurogenesis, microenvironment, and cell death in the adult rat hippocampus. *Developmental neurobiology* 68(5)**,** 575-589. doi: 10.1002/dneu.20600.

Martínez-Téllez, R.I., Hernández-Torres, E., Gamboa, C., and Flores, G. (2009). Prenatal stress alters spine density and dendritic length of nucleus accumbens and hippocampus neurons in rat offspring. *Synapse* 63(9)**,** 794-804. doi: 10.1002/syn.20664.

McCormick, C.M., Smythe, J.W., Sharma, S., and Meaney, M.J. (1995). Sex-specific effects of prenatal stress on hypothalamic-pituitary-adrenal responses to stress and brain glucocorticoid receptor density in adult rats. *Developmental Brain Research* 84(1)**,** 55-61. doi: http://dx.doi.org/10.1016/0165-3806(94)00153-Q.

Michelsen, K.A., van den Hove, D.L., Schmitz, C., Segers, O., Prickaerts, J., and Steinbusch, H.W. (2007). Prenatal stress and subsequent exposure to chronic mild stress influence dendritic spine density and morphology in the rat medial prefrontal cortex. *BMC neuroscience* 8(1)**,** 1. doi: 10.1186/1471-2202-8-107.

Mirescu, C., Peters, J.D., and Gould, E. (2004). Early life experience alters response of adult neurogenesis to stress. *Nature neuroscience* 7(8)**,** 841-846. doi: 10.1038/nn1290.

Monroy, E., Hernández-Torres, E., and Flores, G. (2010). Maternal separation disrupts dendritic morphology of neurons in prefrontal cortex, hippocampus, and nucleus accumbens in male rat offspring. *Journal of Chemical Neuroanatomy* 40(2)**,** 93-101. doi: http://dx.doi.org/10.1016/j.jchemneu.2010.05.005.

Morley-Fletcher, S., Mairesse, J., Soumier, A., Banasr, M., Fagioli, F., Gabriel, C., et al. (2011). Chronic agomelatine treatment corrects behavioral, cellular, and biochemical abnormalities induced by prenatal stress in rats. *Psychopharmacology* 217(3)**,** 301-313. doi: 10.1007/s00213-011-2280-x.

Mueller, B.R., and Bale, T.L. (2008). Sex-specific programming of offspring emotionality after stress early in pregnancy. *The Journal of Neuroscience* 28(36)**,** 9055-9065. doi: http://dx.doi.org/10.1523/JNEUROSCI.1424-08.2008.

Muhammad, A., Carroll, C., and Kolb, B. (2012). Stress during development alters dendritic morphology in the nucleus accumbens and prefrontal cortex. *Neuroscience* 216**,** 103-109. doi: http://dx.doi.org/10.1016/j.neuroscience.2012.04.041.

Murgatroyd, C., Patchev, A.V., Wu, Y., Micale, V., Bockmuhl, Y., Fischer, D., et al. (2009). Dynamic DNA methylation programs persistent adverse effects of early-life stress. *Nat Neurosci* 12(12)**,** 1559-1566. doi: 10.1038/nn.2436.

Murmu, M.S., Salomon, S., Biala, Y., Weinstock, M., Braun, K., and Bock, J. (2006). Changes of spine density and dendritic complexity in the prefrontal cortex in offspring of mothers exposed to stress during pregnancy. *Eur J Neurosci* 24(5)**,** 1477-1487. doi: 10.1111/j.1460-9568.2006.05024.x.

Naninck, E.F., Hoeijmakers, L., Kakava-Georgiadou, N., Meesters, A., Lazic, S.E., Lucassen, P.J., et al. (2015). Chronic early life stress alters developmental and adult neurogenesis and impairs cognitive function in mice. *Hippocampus* 25(3)**,** 309-328. doi: 10.1002/hipo.22374.

O'Malley, D., Dinan, T.G., and Cryan, J.F. (2011). Neonatal maternal separation in the rat impacts on the stress responsivity of central corticotropin-releasing factor receptors in adulthood. *Psychopharmacology* 214(1)**,** 221-229. doi: 10.1007/s00213-010-1885-9.

Ono, M., Kikusui, T., Sasaki, N., Ichikawa, M., Mori, Y., and Murakami-Murofushi, K. (2008). Early weaning induces anxiety and precocious myelination in the anterior part of the basolateral amygdala of male Balb/c mice. *Neuroscience* 156(4)**,** 1103-1110. doi: 10.1016/j.neuroscience.2008.07.078.

Oomen, C.A., Soeters, H., Audureau, N., Vermunt, L., van Hasselt, F.N., Manders, E.M., et al. (2011). Early maternal deprivation affects dentate gyrus structure and emotional learning in adult female rats. *Psychopharmacology* 214(1)**,** 249-260. doi: 10.1007/s00213-010-1922-8.

Oomen, C.A., Soeters, H., Audureau, N., Vermunt, L., van Hasselt, F.N., Manders, E.M., et al. (2010). Severe early life stress hampers spatial learning and neurogenesis, but improves hippocampal synaptic plasticity and emotional learning under high-stress conditions in adulthood. *The journal of Neuroscience* 30(19)**,** 6635-6645. doi: http://dx.doi.org/10.1523/JNEUROSCI.0247-10.2010.

Patin, V., Lordi, B., Vincent, A., and Caston, J. (2005). Effects of prenatal stress on anxiety and social interactions in adult rats. *Developmental Brain Research* 160(2)**,** 265-274. doi: http://dx.doi.org/10.1016/j.devbrainres.2005.09.010.

Rees, S.L., Steiner, M., and Fleming, A.S. (2006). Early deprivation, but not maternal separation, attenuates rise in corticosterone levels after exposure to a novel environment in both juvenile and adult female rats. *Behavioural Brain Research* 175(2)**,** 383-391. doi: http://dx.doi.org/10.1016/j.bbr.2006.09.013.

Rice, C.J., Sandman, C.A., Lenjavi, M.R., and Baram, T.Z. (2008). A novel mouse model for acute and long-lasting consequences of early life stress. *Endocrinology* 149(10)**,** 4892-4900. doi: http://dx.doi.org/10.1210/en.2008-0633.

Roceri, M., Cirulli, F., Pessina, C., Peretto, P., Racagni, G., and Riva, M.A. (2004). Postnatal repeated maternal deprivation produces age-dependent changes of brain-derived neurotrophic factor expression in selected rat brain regions. *Biological Psychiatry* 55(7)**,** 708-714. doi: http://dx.doi.org/10.1016/j.biopsych.2003.12.011.

Romano-Lopez, A., Mendez-Diaz, M., Garcia, F.G., Regalado-Santiago, C., Ruiz-Contreras, A.E., and Prospero-Garcia, O. (2016). Maternal separation and early stress cause long-lasting effects on dopaminergic and endocannabinergic systems and alters dendritic morphology in the nucleus accumbens and frontal cortex in rats. *Dev Neurobiol* 76(8)**,** 819-831. doi: 10.1002/dneu.22361.

Romeo, R.D., Mueller, A., Sisti, H.M., Ogawa, S., McEwen, B.S., and Brake, W.G. (2003). Anxiety and fear behaviors in adult male and female C57BL/6 mice are modulated by maternal separation. *Hormones and Behavior* 43(5)**,** 561-567. doi: http://dx.doi.org/10.1016/S0018-506X(03)00063-1.

Roth, T.L., Lubin, F.D., Funk, A.J., and Sweatt, J.D. (2009). Lasting epigenetic influence of early-life adversity on the BDNF gene. *Biol Psychiatry* 65(9)**,** 760-769. doi: 10.1016/j.biopsych.2008.11.028.

Sadler, T.R., Nguyen, P.T., Yang, J., Givrad, T.K., Mayer, E.A., Maarek, J.M., et al. (2011). Antenatal maternal stress alters functional brain responses in adult offspring during conditioned fear. *Brain Res* 1385**,** 163-174. doi: 10.1016/j.brainres.2011.01.104.

Salm, A.K., Pavelko, M., Krouse, E.M., Webster, W., Kraszpulski, M., and Birkle, D.L. (2004). Lateral amygdaloid nucleus expansion in adult rats is associated with exposure to prenatal stress. *Developmental Brain Research* 148(2)**,** 159-167. doi: http://dx.doi.org/10.1016/j.devbrainres.2003.11.005.

Schulz, K.M., Pearson, J.N., Neeley, E.W., Berger, R., Leonard, S., Adams, C.E., et al. (2011). Maternal stress during pregnancy causes sex-specific alterations in offspring memory performance, social interactions, indices of anxiety, and body mass. *Physiology & Behavior* 104(2)**,** 340-347. doi: http://dx.doi.org/10.1016/j.physbeh.2011.02.021.

Seidel, K., Helmeke, C., Poeggel, G., and Braun, K. (2008). Repeated neonatal separation stress alters the composition of neurochemically characterized interneuron subpopulations in the rodent dentate gyrus and basolateral amygdala. *Dev Neurobiol* 68(9)**,** 1137-1152. doi: 10.1002/dneu.20651.

Son, G.H., Geum, D., Chung, S., Kim, E.J., Jo, J.H., Kim, C.M., et al. (2006). Maternal stress produces learning deficits associated with impairment of NMDA receptor-mediated synaptic plasticity. *J Neurosci* 26(12)**,** 3309-3318. doi: 10.1523/JNEUROSCI.3850-05.2006.

Sowa, J., Bobula, B., Glombik, K., Slusarczyk, J., Basta-Kaim, A., and Hess, G. (2015). Prenatal stress enhances excitatory synaptic transmission and impairs long-term potentiation in the frontal cortex of adult offspring rats. *PloS one* 10(3)**,** e0119407.

St-Cyr, S., and McGowan, P.O. (2015). Programming of stress-related behavior and epigenetic neural gene regulation in mice offspring through maternal exposure to predator odor. *Front Behav Neurosci* 9**,** 145. doi: 10.3389/fnbeh.2015.00145.

Szuran, T.F., Pliška, V., Pokorny, J., and Welzl, H. (2000). Prenatal stress in rats: effects on plasma corticosterone, hippocampal glucocorticoid receptors, and maze performance. *Physiology & behavior* 71(3)**,** 353-362.

Tata, D.A., Markostamou, I., Ioannidis, A., Gkioka, M., Simeonidou, C., Anogianakis, G., et al. (2015). Effects of maternal separation on behavior and brain damage in adult rats exposed to neonatal hypoxia–ischemia. *Behavioural Brain Research* 280**,** 51-61. doi: http://dx.doi.org/10.1016/j.bbr.2014.11.033.

Trujillo, V., Durando, P.E., and Suarez, M.M. (2016). Maternal separation in early life modifies anxious behavior and Fos and glucocorticoid receptor expression in limbic neurons after chronic stress in rats: effects of tianeptine. *Stress* 19(1)**,** 91-103. doi: 10.3109/10253890.2015.1105958.

van der Doelen, R.H., Arnoldussen, I.A., Ghareh, H., van Och, L., Homberg, J.R., and Kozicz, T. (2015). Early life adversity and serotonin transporter gene variation interact to affect DNA methylation of the corticotropin-releasing factor gene promoter region in the adult rat brain. *Dev Psychopathol* 27(1)**,** 123-135. doi: 10.1017/S0954579414001345.

Van Waes, V., Enache, M., Dutriez, I., Lesage, J., Morley-Fletcher, S., Vinner, E., et al. (2006). Hypo-response of the hypothalamic-pituitary-adrenocortical axis after an ethanol challenge in prenatally stressed adolescent male rats. *Eur J Neurosci* 24(4)**,** 1193-1200. doi: 10.1111/j.1460-9568.2006.04973.x.

Wang, A., Nie, W., Li, H., Hou, Y., Yu, Z., Fan, Q., et al. (2014). Epigenetic Upregulation of Corticotrophin-Releasing Hormone Mediates Postnatal Maternal Separation-Induced Memory Deficiency. *PLoS ONE* 9(4)**,** e94394. doi: 10.1371/journal.pone.0094394.

Wang, H., Meyer, K., and Korz, V. (2013a). Stress induced hippocampal mineralocorticoid and estrogen receptor beta gene expression and long-term potentiation in male adult rats is sensitive to early-life stress experience. *Psychoneuroendocrinology* 38(2)**,** 250-262. doi: 10.1016/j.psyneuen.2012.06.004.

Wang, Q., Li, M., Du, W., Shao, F., and Wang, W. (2015). The different effects of maternal separation on spatial learning and reversal learning in rats. *Behavioural Brain Research* 280**,** 16-23. doi: http://dx.doi.org/10.1016/j.bbr.2014.11.040.

Wang, X., Meng, F.-S., Liu, Z.-Y., Fan, J.-M., Hao, K., Chen, X.-Q., et al. (2013b). Gestational Hypoxia Induces Sex-Differential Methylation of Crhr1 Linked to Anxiety-like Behavior. *Molecular Neurobiology* 48(3)**,** 544-555. doi: 10.1007/s12035-013-8444-4.

Weaver, I.C., Cervoni, N., Champagne, F.A., D'Alessio, A.C., Sharma, S., Seckl, J.R., et al. (2004). Epigenetic programming by maternal behavior. *Nat Neurosci* 7(8)**,** 847-854. doi: 10.1038/nn1276.

Workel, J.O., Oitzl, M.S., Fluttert, M., Lesscher, H., Karssen, A., and de Kloet, E.R. (2001). Differential and age-dependent effects of maternal deprivation on the hypothalamic-pituitary-adrenal axis of brown norway rats from youth to senescence. *J Neuroendocrinol* 13(7)**,** 569-580.

Xiong, G.J., Yang, Y., Wang, L.P., Xu, L., and Mao, R.R. (2014). Maternal separation exaggerates spontaneous recovery of extinguished contextual fear in adult female rats. *Behav Brain Res* 269**,** 75-80. doi: 10.1016/j.bbr.2014.04.015.

Yang, J., Hou, C., Ma, N., Liu, J., Zhang, Y., Zhou, J., et al. (2007). Enriched environment treatment restores impaired hippocampal synaptic plasticity and cognitive deficits induced by prenatal chronic stress. *Neurobiology of Learning and Memory* 87(2)**,** 257-263. doi: http://dx.doi.org/10.1016/j.nlm.2006.09.001.

Yang, J., Li, W., Liu, X., Li, Z., Li, H., Yang, G., et al. (2006). Enriched environment treatment counteracts enhanced addictive and depressive-like behavior induced by prenatal chronic stress. *Brain research* 1125(1)**,** 132-137. doi: 10.1016/j.brainres.2006.10.028.

Zalosnik, M.I., Pollano, A., Trujillo, V., Suarez, M.M., and Durando, P.E. (2014). Effect of maternal separation and chronic stress on hippocampal-dependent memory in young adult rats: evidence for the match-mismatch hypothesis. *Stress* 17(5)**,** 445-450. doi: 10.3109/10253890.2014.936005.

Zhu, X., Li, T., Peng, S., Ma, X., Chen, X., and Zhang, X. (2010). Maternal deprivation-caused behavioral abnormalities in adult rats relate to a non-methylation-regulated D2 receptor levels in the nucleus accumbens. *Behavioural Brain Research* 209(2)**,** 281-288. doi: http://dx.doi.org/10.1016/j.bbr.2010.02.005.

Zohar, I., and Weinstock, M. (2011). Differential Effect of Prenatal Stress on the Expression of Cortiocotrophin-Releasing Hormone and its Receptors in the Hypothalamus and Amygdala in Male and Female Rats. *Journal of Neuroendocrinology* 23(4)**,** 320-328. doi: 10.1111/j.1365-2826.2011.02117.x.

Zuena, A.R., Mairesse, J., Casolini, P., Cinque, C., Alema, G.S., Morley-Fletcher, S., et al. (2008). Prenatal restraint stress generates two distinct behavioral and neurochemical profiles in male and female rats. *PLoS One* 3(5)**,** e2170. doi: 10.1371/journal.pone.0002170.
